# The interplay of the lncRNA AFAP1-AS1 and the soluble immune checkpoint proteins sPD-1/sPD-L1 in breast cancer: tumor–immune cross-talk

**DOI:** 10.1042/BSR20250198

**Published:** 2026-10-05

**Authors:** Sherihan G. AbdelHamid, Ghadir S. EL-Housseiny, Hany M. AbdelAziz, Amany M. Kamal

**Affiliations:** 1 Department of Biochemistry and Molecular Biology, Faculty of Pharmacy, Ain Shams University, Cairo, Egypt; 2 Department of Microbiology and Immunology, Faculty of Pharmacy, Ain Shams University, Cairo, Egypt; 3 Department of Clinical Oncology, Faculty of Medicine, Ain Shams University, Cairo, Egypt

**Keywords:** biomarkers, breast cancers, large intervening non-coding RNA

## Abstract

Breast cancer (BC) remains a major health issue worldwide. Immuno-oncology has recently garnered much attention with unprecedented bench-to-bedside revolutions in cancer diagnosis and treatment. The PD-1/PD-L1 pathway is an adaptive mechanism in cancer immune escape that can be regulated epigenetically via long non-coding RNAs (lncRNAs). In the present study, we sought to investigate the potential clinical utility of the circulating lncRNA AFAP1-AS1 and soluble immune checkpoint proteins sPD-1 and sPD-L1 as liquid biopsy biomarkers in BC patients. Serum expression levels of AFAP1-AS1 were quantified via qRT‒PCR in a cohort comprising 90 BC patients (45 metastatic and 45 nonmetastatic) and 20 healthy controls, while serum sPD-1 and sPD-L1 levels were determined by ELISA. Computational bioinformatics approaches were employed to investigate molecular interactions among AFAP1-AS1, PD-1, and PD-L1 and to assess their functional roles in oncogenesis. Serum AFAP1-AS1, sPD-1, and sPD-L1 levels exhibited significant elevation in BC patients compared with controls, with further increases observed in metastatic versus non-metastatic cases. These biomarkers demonstrated significant association with positive lymph node involvement (*P* < 0.05). AFAP1-AS1 showed significant correlations with sPD-1 and sPD-L1, and their molecular interactions were demonstrated through *in silico* predictive analyses. ROC analysis revealed robust diagnostic and prognostic discriminatory potential. In conclusion, the present study underscores the clinical potential of the circulating lncRNA AFAP1-AS1, sPD-1, and sPD-L1 as promising non-invasive biomarkers in BC and may collectively reflect a coordinated immune evasion axis. The interplay between epigenetic modifications and immune checkpoint pathways represents a novel therapeutic target for enhancing antitumor immunity and halting metastasis.

## Background

Breast cancer (BC) is the most ubiquitous malignancy in females and the second most widespread cancer globally [1]. Its development and progression are mediated by genetic and epigenetic alterations [2,3]. There is an imperative necessity for specific and sensitive noninvasive biomarkers that can augment traditional modalities to improve BC clinical management. Liquid biopsy has been regarded as a breakthrough in the era of clinical oncology. It involves the detection of circulating tumor cells, exosomes, circulating tumor DNA, and other soluble biomarkers that reflect the tumor landscape while using minimally invasive procedures [4,5]. The data obtained are currently being studied to aid in cancer diagnosis, screening, prediction of prognosis, and guiding therapy for different tumor types.

Among these biomarkers, soluble immune checkpoints have attracted considerable attention [6]. Immune checkpoints play an imperative role in immune regulation and tumorigenesis [7]. They act as negative regulators to avert excess damage to peripheral tissues upon continued immune-cell activation by inhibiting the production of cytotoxic signals by immune cells [8], thereby regulating the duration and intensity of the immune response [9]. Among the various immune checkpoint proteins studied in oncology, programmed cell death-1 (PD-1) and programmed cell death ligand-1 (PD-L1) are of substantial interest [10,11].

PD-1, or CD279, is a transmembrane glycoprotein (type I) encoded by the PDCD1 gene expressed on lymphoid cells, including natural killer (NK) cells, T and B cells [12,13]. It consists of a transmembrane region, an intracellular tail, and an immunoglobulin V-type extracellular domain [12]. PD-1 plays a pivotal role in regulating immune responses via modulating the activity of antigen-specific T cells and regulatory T cells [14]. PD-L1, also known as CD274, binds to PD-1 to prevent prolonged immune activation via the delivery of a coinhibitory signal [11,15]. PD-L1 ligation triggers PD-1-mediated intracellular signaling, resulting in the suppression of the RAS-MEK-ERK and PI3K-AKT-mTOR pathways. This, in turn, results in the restriction of T-cell growth, differentiation, cell division, cell cycle progression, and survival [14]. It has also been shown that PD-1 expression can negatively influence the cytotoxic capacity of NK cells and cytokine production and has inhibitory effects on monocytes and macrophages [16–18].

The PD-1/PD-L1 pathway is regarded as an adaptive mechanism of cancer cells for immune resistance [19,20]. PD-L1 to PD-1 in tumors induces inhibition of cytotoxic T lymphocyte effector function and generates an immunosuppressive environment, permitting tumor cells to evade immune-mediated destruction [8]. PD-L1 may also be implicated in cancer initiation, development, progression, and treatment resistance through inducing pro-survival signals within tumor cells [21]. Blocking this pathway with immune checkpoint inhibitors can reactivate antitumor immune activity and immune surveillance [19,20,22].

The detection of soluble immune checkpoints in serum samples has revealed a novel paradigm for research [23–25]. Soluble PD-1 (sPD-1) is a monomeric protein produced via alternative splicing or proteolytic cleavage. Four splice variants have been detected; PD-1△3 may be considered soluble PD-1, as it can bind to its ligands [14,24]. Three mechanisms may play a role in the appearance of sPD-L1 in the circulation: proteolytic cleavage from the surface of PD-L1-expressing cells [26], release of PD-L1 from extracellular vesicles [27], or alternatively spliced transcripts [28]. Elevated serum sPD-1 and sPD-L1 levels in several types of cancers were shown to promote immunosuppression and are hence regarded as unfavorable prognostic factors [23,29–31]. However, the levels of these soluble molecules have not been adequately studied in the serum of patients with BC.

Regulation of the PD-1/PD-L1 axis can occur at the epigenetic, genomic, transcriptional, or translational level and is mediated by several molecules, including microRNAs, long noncoding RNAs (lncRNAs), and cytokines [32,33]. Investigating these underlying mechanisms may help in the development of effective directed anticancer therapies and can guide the genetic manipulation of these pathways. LncRNAs belong to a large class of noncoding RNAs >200 nucleotides in length that regulate gene expression at the epigenetic level [34–37]. Among the newly discovered lncRNAs, actin fiber-associated protein 1-antisense RNA1 (AFAP1-AS1), the antisense of AFAP1, acts as an adaptor protein by linking actin filaments to signaling proteins [38]. Aberrant expression of AFAP1-AS1 has been found to be associated with tumor development and progression [39–41].

While several studies have implicated lncRNAs in the regulation of immune checkpoint pathways through ceRNA networks and transcriptional co-regulation, the specific molecular mechanisms by which AFAP1-AS1 may interact with PD-1/PD-L1 signaling in BC remain incompletely characterized. The present study aimed to evaluate the circulating serum levels of the lncRNA AFAP1-AS1, sPD-1, and sPD-L1 in metastatic and non-metastatic BC patients compared with those in healthy controls as non-invasive biomarkers and their associations with clinicopathological characteristics. In addition, *in silico* analysis was employed to investigate molecular interactions among AFAP1-AS1, PD-1, and PD-L1 and to assess their functional roles in oncogenesis.

## Materials and methods

### *In silico* analysis

#### Characterization

Characterization of AFAP1-AS1 through the HUGO Gene Nomenclature Committee (HGNC) https://www.genenames.org/, National Center for Biotechnology Information (NCBI) https://www.ncbi.nlm.nih.gov/, NCBI Genome Data Viewer (GDV), and Ensembl databases https://www.ensembl.org/index.html is shown in Table 1.

**Table 1:** *AFAP1-AS1* gene characterization

Gene symbol	*CCDC144NL-AS1*
**Genomic region**	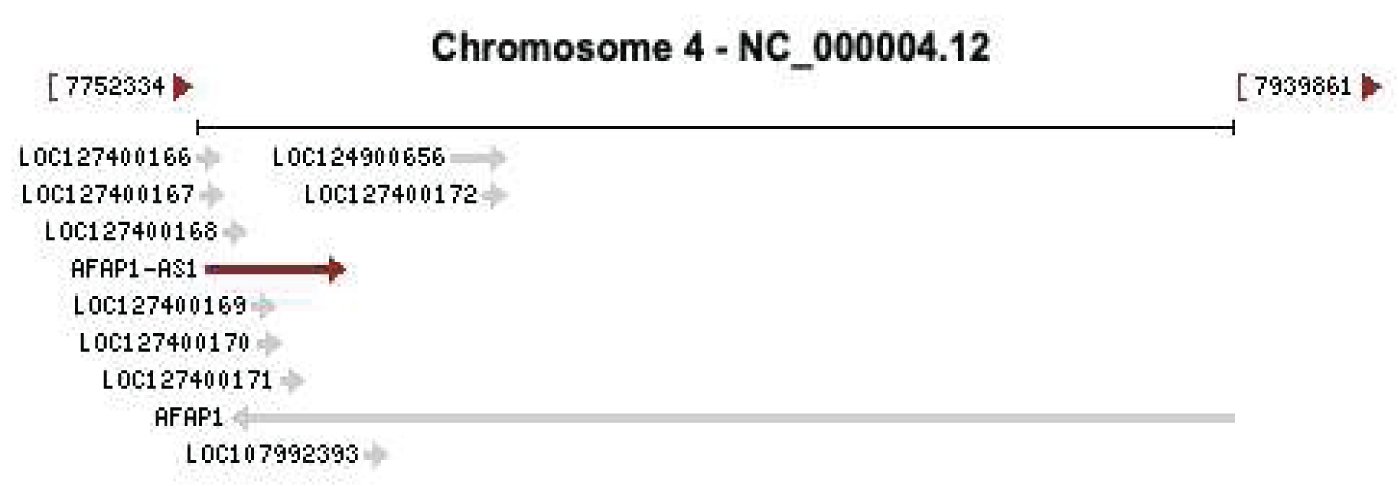
**HGNC**	https://www.genenames.org/data/gene-symbol-report/#!/hgnc_id/HGNC:28141
**Chromosomal location**	4p16.1
**Ensembl gene**	ENSG00000272620
**NCBI Gene ID**	440416
**GDV-NCBI**	https://www.ncbi.nlm.nih.gov/genome/gdv/browser/gene/?id=440416

#### lncRNA AFAP1-AS1 expression in cancer

lncRNA AFAP1-AS1 expression was evaluated across various cancer types via data retrieved from validated experimental results in publications or predicted outcomes, as provided by the LncRNADisease v3.0 database (http://www.rnanut.net/lncrnadisease/index.php/home/detail/LDA0004175). The network analysis was conducted via the cytoHubba plugin within Cytoscape, highlighting the top 10 nodes ranked by maximal clique centrality (MCC).

#### ENCORI Pan-Cancer Analysis Platform

The fold change in the expression levels of the lncRNA AFAP1-AS1 in BC and control samples was assessed via the ENCORI Pan-Cancer Analysis Platform (https://rnasysu.com/encori/panCancer.php) across 32 types of cancers.

#### TCGA database analysis

##### Data preprocessing

The present study utilized RNA-Seq data, survival data, and clinical information obtained from the Cancer Genome Atlas (TCGA) database through UCSC Xena platform. The standardization of data was performed using DESeq2. The retrieved dataset (TCGA-BRCA) comprised 1227 samples from both normal and tumor patients with expression profiles of 60 660 genes accessible on TCGA database (https://gdc-hub.s3.us-east-1.amazonaws.com/download/TCGA-BRCA.star_counts.tsv.gz). Additionally, survival data (https://gdc-hub.s3.us-east-1.amazonaws.com/download/TCGA-BRCA.survival.tsv.gz) and clinical information (https://gdc-hub.s3.us-east-1.amazonaws.com/download/TCGA-BRCA.clinical.tsv.gz) of TCGA BRCA were retrieved from UCSC Xena for further analysis. Data analysis of TCGA-BRCA was initiated by integrating RNA-Seq data and phenotype data using TCGA-BRCA sample IDs to distinguish normal samples designated as “Solid Tissue Normal”, tumor samples designated as “Primary Tumor”, and metastatic samples designated as “Metastatic”. The curated dataset comprised 1226 TCGA-BRCA samples categorized as normal (*n* = 113), tumor (*n* = 1106), and metastatic samples (*n* = 7). Furthermore, samples were further stratified into high AFAP1-AS1 (*n* = 632) and low AFAP1-AS1 (*n* = 594) based on median expression levels for subsequent analysis. All manually curated data is provided in Supplementary Table S1.

##### Identifying TCGA BRCA differential expression signature

To assess differential gene expression of AFAP1-AS1 across TCGA-BRCA samples, R packages “ensembldb v. 2.28.1” and “AnnotationHub v.3.12.0” were used to convert Ensembl IDs for all genes listed in the TCGA BRCA gene expression dataset. Next, differentially expressed genes (DEGs) were determined using R package “limma v.3.60.4” based on specific criteria: false discovery rate (FDR) ≤ 0.05, *P*-value ≤ 0.05; then log fold change was calculated via Benjamini–Hochberg (BH) method for *P*-adjusted scores. The resulting DEGs were then visualized as heatmaps, box plots, and volcano plots generated using R packages “ggplot2 v.3.5.1”, “heatmap v.1.0.12”, and “EnhancedVolcano v.1.22.0.

##### Gene set enrichment analysis

To characterize molecular functions and pathways associated with DEGs in TCGA BRCA, gene set enrichment analysis (GSEA) was conducted using Gene Ontology (GO) database utilizing R package “clusterProfiler v.4.12.5”, which performed gene set enrichment with maximum gene set at 800 (*P* ≤ 0.05). R package “DOSE v.3.30.5” was used for plotting profiles of DEGs for each group, including TCGA-BRCA samples and high/low AFAP-AS1 TCGA-BRCA samples, with the top 50 DEGs per group were included in the analysis.

##### Pearson’s correlation analysis

Correlations between AFAP1-AS1, PD-1, and PD-L1 in TCGA BRCA were evaluated using R package “corrplot v.0.95”. TCGA BRCA dataset was divided into two different sets where correlation is calculated in TCGA-BC samples as follows (AFAP1-AS1 and PD-1, AFAP1-AS1, and PD-L1). Scatter plots were generated for each of the two studied groups across two conditions (TCGA-BRCA samples and high AFAP-1AS1 TCGA-BRCA samples) using R package “ggplot2 v.3.5.1” (*P* ≤ 0.05).

##### Survival analysis

Next, survival analysis of TCGA-BRCA patients was performed, where samples were categorized into high and low AFAP1-AS1 based on median gene expression level. Then, R packages “survival v.3.5” and “survminer v.0.4.9”, Kaplan–Meier (KM) plots for overall survival (OS) and progression-free survival (PFS) were conducted through univariate Cox regression analysis to calculate the probability of survival.

#### Prediction of lncRNA–protein interactions via RPISeq

To assess the potential physical interactions between the lncRNA AFAP1-AS1 and the immune checkpoint proteins sPD-1 and sPD-L1, *in silico* prediction was performed via the RNA–protein interaction prediction (RPISeq) tool http://pridb.gdcb.iastate.edu/RPISeq. FASTA sequences of AFAP1-AS1 and the amino acid sequences of PD-1 and PD-L1 were retrieved from publicly available databases. These sequences were submitted to RPISeq, which uses two machine learning classifiers—random forest (RF) and support vector machine (SVM)—to calculate interaction probabilities.

#### Interaction of the lncRNA AFAP1-AS1 with miRNAs via Enricher

To explore the regulatory interactions of the lncRNA AFAP1-AS1, we identified potential target miRNAs via Enrichr (https://maayanlab.cloud/Enrichr/) through April 2025. The TargetScan microRNA 2017 dataset, which provides predicted miRNA‒mRNA interactions on the basis of seed region complementarity and evolutionary conservation, was selected. In the Enrichr interface, we navigated to the “Target Scan” section and input a curated list of miRNAs previously associated with AFAP1-AS1 (obtained from the literature and predicted databases).

#### Functional enrichment analysis

Protein‒RNA interaction predictions with supporting experimental data were searched via the RNAct database (https://rnact.crg.eu). Pathways associated with PD-1 and PDL-1 were retrieved from the KEGG database (https://www.genome.jp/kegg/kegg1.html). The STRING database (https://string-db.org) was utilized to study PPI networks and perform functional enrichment analysis. PD-1 and PD-L1 signaling pathway data were retrieved from the Reactome database (https://reactome.org).

### Subjects

The current study comprised 90 females with histopathologically proven invasive BC, alongside 20 age-matched healthy control subjects. BC patients were recruited from Ain Shams University Hospitals, Clinical Oncology Department, Egypt, and were classified into two groups. The first group included 45 treatment-naïve patients who had not received radiotherapy or chemotherapy and had no evidence of cancer at other sites at the time of diagnosis, whereas the second group included 45 BC patients suffering from metastatic disease. Metastasis was confirmed by bone scanning, chest radiography, and liver ultrasound. The exclusion criteria included subjects suffering from any inflammatory conditions, immunological disorders, cardiovascular diseases, or diabetes. The clinical and pathological characteristics, including tumor size, histological type, hormone receptor status (ER, PR, and HER-2/neu), nodal involvement, and Ki-67 expression, were extracted from medical records.

Clinical and pathological staging were determined following the guidelines of the American Joint Committee on Cancer (AJCC) Cancer Staging Manual [42]. The TNM classification was established by integrating tumor size (T), regional lymph node involvement (N), and the presence or absence of distant metastasis (M). These parameters were subsequently combined to assign each patient an overall anatomic disease stage, designated as Stage I, II, III, and Stage IV.

According to the American Society of Clinical Oncology/College of American Pathologists (ASCO/CAP), BCs with <1% ER or PR expression are considered hormone receptor–negative tumors [43]. HER2 status was classified as negative (IHC score 0, 1+, and 2+ not amplified) or positive (IHC score 3 or HER2 amplified by FISH) according to ASCO/CAP guideline recommendations for HER2 testing in BC [44]. Molecular subtype classification of BCs was performed via immunohistochemical surrogate markers according to the 19th International St. Gallen consensus [45], which divides tumors into four main subtypes: luminal A, luminal B, HER2‐positive, and triple‐negative BC (TNBC). ER-positive, HER2-negative tumors with high PR expression and low Ki-67 are classified as Luminal A. While Luminal B are either defined as ER-positive, HER2-negative, and either high Ki-67, high or low PR expression (Luminal B-like HER2-negative), or luminal B-like HER2-positive if they are ER-positive, HER2-positive with any Ki-67 or PR expression. HER2-positive tumors are ER/PR-negative and HER2-positive. ER, PR, and HER2-negative tumors are classified as TNBC.

Informed written consent was obtained from all enrolled subjects after the purpose of the experiment was explained, and the study was approved by the Faculty of Pharmacy Ethical Research Committee at Ain Shams University (32/2021). The study was carried out in accordance with the ethical guidelines of the Helsinki Declaration.

### Blood samples

Venous blood samples (approximately 10 ml) were collected from all the study participants. For serum preparation, samples were centrifuged for 10 min at 15 000 × ***g*** and then at 20 000 × ***g*** for 3 min. Sera were then divided into 3 aliquots for RNA extraction and enzyme-linked immunosorbent (ELISA) assays of sPD-1 and sPD-L1, and then stored at −80°C.

### Serum lncRNA AFAP1-AS1 assay

Total RNA was extracted from 200 μl of each serum sample via a miRNeasy mini kit (Qiagen, Hilden, Germany) as per the manufacturer’s protocol. The quality and quantity of the extracted RNA were evaluated via a NanoDrop 2000 spectrophotometer (Thermo Scientific, U.S.A.).

Reverse transcription was carried out via a High Capacity cDNA Reverse Transcription Kit (Applied Biosystems, Foster City, California) according to the manufacturer’s instructions. Briefly, 1 μg of RNA was added to a 20-μl final reaction mixture and incubated for 10 min at 25°C, 120 min at 37°C, or 5 min at 85°C. The obtained complementary DNA (cDNA) was stored at −20°C.

The expression level of LncRNA AFAP1-AS1 was measured by quantitative real-time polymerase chain reaction (qRT‒PCR) carried out on a Rotor Gene Q RT‒PCR cycler utilizing a specific TaqMan^™^ Assays Kit (Assay ID Hs00953441_s1; Applied Biosystems, Foster City, California) with a TaqMan^™^ Universal Master MiX II (catalog no. 4440043; Applied Biosystems, Foster City, California) according to the protocol provided by the manufacturer under PCR cycling conditions: 10 min at 95°C, followed by 40 cycles of 15 s at 95°C and then 1 min at 60°C. The expression level of the lncRNA was normalized to that of the housekeeping gene GAPDH as an endogenous control and analyzed via the 2^−ΔΔCt^ method.

### Determination of serum sPD-1 and sPD-L1 levels by ELISA

The serum levels of sPD-1 and sPD-L1 was measured using ELISA kits (catalog numbers E6493Hu and E4829Hu, respectively, Bioassay Technology Laboratory, England/China). ELISA procedures were conducted using the Hyprep automated ELISA system (Hyperion Inc., Miami, FL) following the manufacturer’s instructions. Briefly, 40 μl sample was added to sample wells with 10 μl of anti-sPD1 or anti-sPD-L1 antibody, then 50 μl streptavidin-HRP was added, incubated for 60 min at 37°C, followed by washing, then 50 μl of each substrate solution was added, incubated for 10 min at 37°C in the dark. Stop solution was then added and the optical density was measured at 450 nm. The serum levels of sPD-1 and sPD-L1 were calculated via interpolation from calibration curve constructed using standard solutions that were prepared by serially diluting the standard stock solution (32 ng/ml) 1:2 with standard diluent.

### Statistical analysis

#### Statistical data analysis

Numerical data were explored for normality using Kolmogrov–Smirnov test and the Shapiro‒Wilk test. Normally distributed numerical variables are presented as the means and standard deviations (SDs), whereas nonnormally distributed variables are presented as medians and interquartile ranges (IQR). The Mann‒Whitney U test was used to compare between-group differences. Categorical variables are presented as numbers and percentages, and intergroup differences were compared via the Pearson chi-square test or Fisher’s exact test. Comparisons of more than two groups were performed via analysis of variance for normally distributed variables and the Kruskal‒Wallis test for nonnormally distributed variables. Correlations were tested via Spearman’s rank correlation (ρ). Receiver operating characteristic (ROC) curve analysis was employed to evaluate the diagnostic and predictive value of the studied biomarkers. Analysis of the data was carried out via IBM^©^ SPSS^©^ Statistics version 26 (IBM^©^ Corp., Armonk, NY).

#### Internal cross-validation analysis

To assess the robustness of our findings, we performed a k-fold (5-fold) cross-validated ROC analysis across three distinct clinical comparisons. We examined the predictive/diagnostic value of the markers in discriminating between cases and controls, discriminating between patients with metastatic or non-metastatic diseases, and discriminating between patients with distant or contralateral-side metastasis. The outcome variable was “Breast Carcinoma”, “Metastatic Disease”, or “Distant Metastasis”, for the three analyses, respectively. The binary outcome variables were coded as “0 = absent”, “1 = present”. Continuous predictors included the expression levels of AFAP1-AS1, sPD1, and sPDL1. The reference category for the categorical covariate “Molecular Subtype” was the “Triple Negative Subtype” (coded as 0). The “Luminal A”, “Luminal B”, and “Triple Positive” subtypes were coded as 1, 2, or 3, respectively.

For each analysis, performance metrics, including area under the curve (AUC), optimal cutoff, sensitivity, and specificity, were calculated with 95% confidence intervals (CIs) derived from the distribution across folds. K-fold cross-validated ROC analysis was performed with adjustment for the categorical covariate “Molecular subtype” in the case of BC patients in order to evaluate the predictive performance of each biomarker individually and in combination. All analyses were conducted using Python version 3.10 and the following libraries: `scikit-learn` (version 1.2.2) for machine learning and cross-validation, `matplotlib` (version 3.7.1) for graphical visualization, and `pandas` (version 2.0.0) for data management.

The analysis followed a five-step procedure. First, the complete dataset was partitioned using stratified 5-fold cross-validation implemented via the `StratifiedKFold` class from `scikit-learn`. Stratification ensured that the proportion of patients with and without distant metastasis was preserved in every fold, which is critical for stable performance estimation when the outcome prevalence is imbalanced. Second, for each of the five iterations, the model was trained on four folds (80% of the data) and tested on the remaining fold (20%). To adjust for potential confounding, all models run on the cases group included the “Molecular Subtype” as a covariate regardless of whether they also contained one of the continuous predictors or all three. Third, for every test fold, we used the fitted logistic regression model (implemented with `LogisticRegression`, solver =`liblinear`) to compute predicted probabilities of distant metastasis for each patient in that fold. From these probabilities, we derived the ROC curve and AUC using the functions `roc_curve` and `auc` from `sklearn.metrics`. The optimal probability cutoff for each fold was determined by maximizing the Youden index (J = sensitivity + specificity − 1). Fourth, after completing the five folds, we calculated the mean AUC, mean optimal cutoff, mean sensitivity, and mean specificity by averaging the respective values across the five folds. To express the uncertainty around these point estimates, we constructed 95% confidence intervals (CIs*) using the percentile method on the distribution of the five-fold-specific estimates (i.e. the 2.5th and 97.5th percentiles). Fifth, the mean ROC curve across the five folds was obtained by interpolating the true positive rate (TPR) to a common grid of false positive rates (FPR) from 0 to 1, after which the across-fold mean TPR was plotted against the FPR grid. A confidence band around the mean ROC curve was constructed by calculating, at each FPR point, the mean TPR ± 1.96 times the standard error of the TPR across the five folds. All graphical outputs were generated with `matplotlib`, and results were exported to CSV files for tabulation.

This approach provides a rigorous, non-optimistic estimate of the models’ discriminatory ability because each prediction is made on a fold of data that was not used for training, thereby mimicking independent validation. The 95% CIs for all performance metrics (AUC, cutoff, sensitivity, and specificity) give a direct measure of the stability and generalizability of our findings. The adjustment for “Molecular Subtype” in every model involving diseased cases ensures that the estimated predictive performance of the biomarkers is independent of this potentially confounding categorical variable.

## Results

The clinicopathological characteristics of the BC cohort are shown in Table 2. The mean age of the BC patients was 53.7 ± 9.9 years (52.7 ± 9.2 for the nonmetastatic patients and 54.6 ± 10.3 for the metastatic patients), whereas it was 50.2 ± 3.2 years for the control subjects. In terms of hormone receptor status, 86.7% of the patients were ER positive, 88.9% were PR positive, and 84% were HER2/*neu* negative. Luminal A was the most commonly observed molecular subtype. Approximately 71.1% of BC patients had positive lymph node involvement. With respect to tumor size, T1 was observed in 12% of BC patients, T2 in 51.1%, T3 in 23.3%, and T4 in 13.3%. Among the patients with metastatic BC, 35.2% had contralateral BC, whereas 64.8% had distant BC. Positive lymph node involvement and HER2-positive tumors were more frequently observed in metastatic BC patients than in nonmetastatic BC patients.

**Table 2:** Clinicopathological characteristics of BC cohort

Clinicopathological parameters	Mean ± SD/n (%)			*P*-value
	Breast cancer (*n* = 90)	Nonmetastatic BC (*n* = 45)	Metastatic BC (*n* = 45)	
**Age (years)**	53.7 ± 9.9	52.7 ± 9.2	54.6 ± 10.3	0.162
**BI-RADS class** *BI-RADS 3* *BI-RADS 4* *BI-RADS 5* *BI-RADS 6* *BI-RADS 3–4* *BI-RADS 5–6*	2 (2.2%) 27 (30.0%) 60 (66.7%) 1 (1.1%) 29 (32.2%) 61 (77.8%)	16 (35.6%) 29 (64.4%)	13 (28.9%) 32 (71.1%)	0.49
**T Stage** *T1–T2* *T3–T4*	57 (63.3%) 33 (36.3%)	31 (68.9%) 14 (31.1%)	26 (57.8%) 19 (42.2%)	0.27
**LN** *N0* *N1–N2*	26 (28.9%) 64 (71.1%)	24 (53.3%) 21 (46.7%)	13 (28.9%) 32 (71.1%)	0.018^*^
**ER** *Negative* *Positive*	12 (13.3%) 78 (86.7%)	8 (17.8%) 37 (82.2%)	4 (8.9%) 41 (91.1%)	0.215
**PR** *Negative* *Positive*	10 (11.1%) 80 (88.9%)	6 (13.3%) 39 (86.7%)	4 (8.9%) 41 (91.1%)	0.5
**HER2/neu** *Negative* *Positive*	84 (88.9%) 6 (6.7%)	45 (100%) 0 (0%)	39 (86.7%) 6 (13.3%)	0.01^*^
**Molecular subtype** *Luminal A* *Luminal B* *HER2-positive* *TNBC*	43 (47.7%) 33 (36.6%) 5 (5.55%) 9 (10%)	11 (24.4%) 27 (60%) 5 (11.1%) 2 (4.44%)	32 (71.1%) 6 (13.3%) 0 (0%) 7 (15.5%)	-

BC, breast cancer; BI-RADS, Breast Imaging Reporting and Data System; T, tumor size; LN, lymph node; ER, estrogen receptor; PR, progesterone receptor; HER2, human epidermal growth factor receptor 2; TNBC, triple-negative breast cancer; SD, standard deviation.

* Statistically significant at *P* ≤ 0.05.

### Serum lncRNA AFAP1-AS1 relative expression, sPD-1, and sPD-L1 levels in BC patients and controls

The fold change expression levels of LncRNA AFAP1-AS1 in BC and control samples were assessed using the ENCORI Pan-Cancer Analysis Platform (https://rnasysu.com/encori/panCancer.php) across 32 types of cancers (FDR 0.0042, *P* = 0.017) (Figure 1A). LncRNA AFAP1-AS1 expression was evaluated across various cancer types, using data retrieved from validated experimental results in publications or predicted outcomes, as provided by the LncRNADisease v3.0 database (http://www.rnanut.net/lncrnadisease/index.php/home/detail/LDA0004175). The network analysis was conducted using the cytoHubba plugin within Cytoscape, highlighting the top 10 nodes ranked by MCC. In the generated network, LncRNA AFAP1-AS1 is represented as the red central circle, while the connections are depicted by gray lines, showcasing its associations with the top 10 causal diseases presented as yellow circles (Figure 1B).

**Figure 1:**
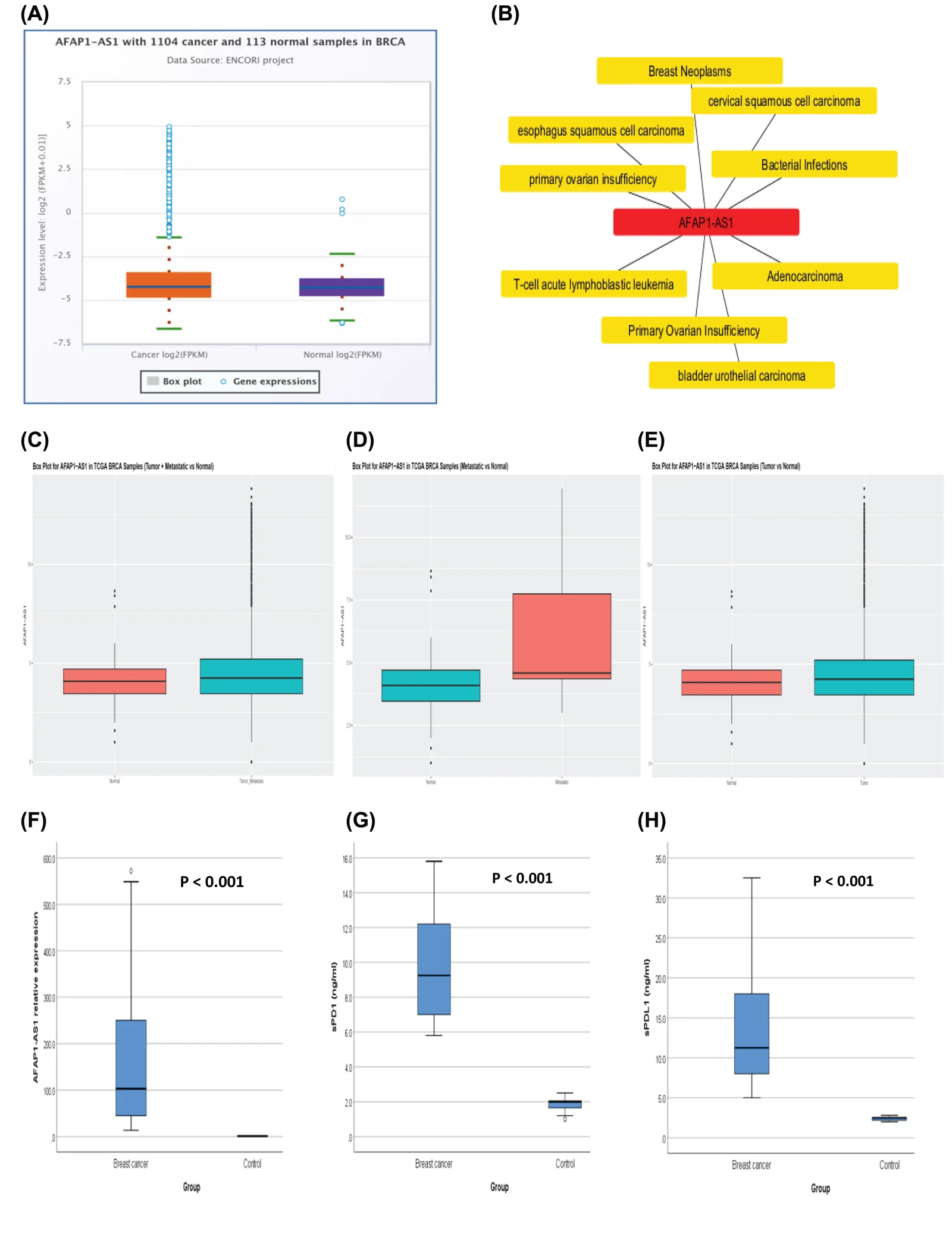
Serum AFAP1-AS1 relative expression, sPD-1, and sPD-L1 levels in BC patients and controls (**A**) The fold change expression levels of LncRNA AFAP1-AS1 in BC and control samples were assessed using the ENCORI Pan-Cancer Analysis Platform (https://rnasysu.com/encori/panCancer.php) across 32 types of cancers. (**B**) The network analysis was conducted using the cytoHubba plugin within Cytoscape, highlighting the top 10 nodes ranked by MCC. In the generated network, LncRNA AFAP1-AS1 is represented as the red central circle, while the connections are depicted by gray lines, showcasing its associations with the top 10 causal diseases presented as yellow circles. (**C**) Box plot for AFAP1-AS1 in TCGA-BRCA samples (tumor + metastatic versus normal). (**D**) Box plot for AFAP1-AS1 in TCGA-BRCA samples (metastatic versus normal). (**E**) Box plot for AFAP1-AS1 in TCGA-BRCA samples (tumor versus normal). (**F**) Box plot illustrating the relative expression of the serum lncRNA AFAP1-AS1 in patients with BC (*n* = 90) and controls (*n* = 20). (**G**) Box plot illustrating serum sPD-1 levels in patients with BC (*n* = 90) and controls (*n* = 20). (**H**) Box plot illustrating serum sPD-L1 levels in patients with BC (*n* = 90) and controls (*n* = 20). The interquartile range in the boxplot (F–H) is represented by a box, and the median is represented by a line inside the box (Mann‒Whitney test). The minimum and maximum values are represented by error bars.

Additionally, TCGA-BRCA samples (*n* = 1227) were used for calculating DEGs across normal (*n* = 113), tumor (*n* = 1106), and metastatic (*n* = 7) tissue samples. Using pre-identified criteria, DEGs were calculated for TCGA-BRCA samples where 22 831 genes were differentially expressed. Box plots were also done to identify AFAP1-AS1 expression across different tumor tissue categories, including TCGA-BRCA samples (tumor/metastatic versus normal) (FC = 4.52, *P* ≤ 0.01) (Figure 1C), TCGA-BRCA samples (tumor versus normal) (FC = 1.70, *P* ≤ 0.01) (Figure 1D), TCGA-BRCA samples (metastatic versus normal) (FC = 1.69, *P* ≤ 0.01) (Figure 1E).

In alignment with the *in silico* analysis, the relative expression of AFAP1-AS1 in the present study cohort was significantly elevated in the patients with BC compared with control subjects (*P* < 0.0001) (Table 3 and Figure 1F). In addition, serum sPD-1 and sPD-L1 levels were significantly higher in BC patients than in controls (*P* < 0.0001) (Table 3, Figure 1G,H, respectively).

**Table 3:** LncRNA AFAP1-AS1 expression, sPD-1, and sPD-L1 levels in BC patients and controls

Variable	Breast cancer (*n* = 90)		Control (*n* = 20)		U-statistic	Z-statistic	*P* value^†^
	Median	IQR	Median	IQR			
**AFAP1-AS1 expression**	102.95	44.94 to 250.35	1.00	0.99 to 1.10	0.000	−6.977	<0.0001
**sPD-1 (ng/ml)**	9.25	7.00 to 12.20	2.00	1.65 to 2.05	0.000	−6.989	<0.0001
**sPD-L1 (ng/ml)**	11.25	8.00 to 18.00	2.50	2.20 to 2.50	0.000	−6.983	<0.0001

IQR, interquartile range.

† Mann‒Whitney test.

### Serum lncRNA AFAP1-AS1 relative expression, sPD-1, and sPD-L1 levels in metastatic and nonmetastatic BC patients

Utilizing the TCGA-BRCA samples, Figure 2A shows a box plot for TCGA-BRCA samples for lncRNA AFAP1-AS1 (tumor versus metastatic). Results of the study cohort were in line with the *in silico* analysis, where serum AFAP1-AS1 expression was significantly higher in metastatic BC patients than in nonmetastatic patients (*P* < 0.0001) (Table 4, Figure 2B). Compared with nonmetastatic patients, metastatic BC patients also exhibited elevated serum sPD-1 and sPD-L1 levels (*P* < 0.0001), as shown in Table 4 and Figure 2C.

**Figure 2:**
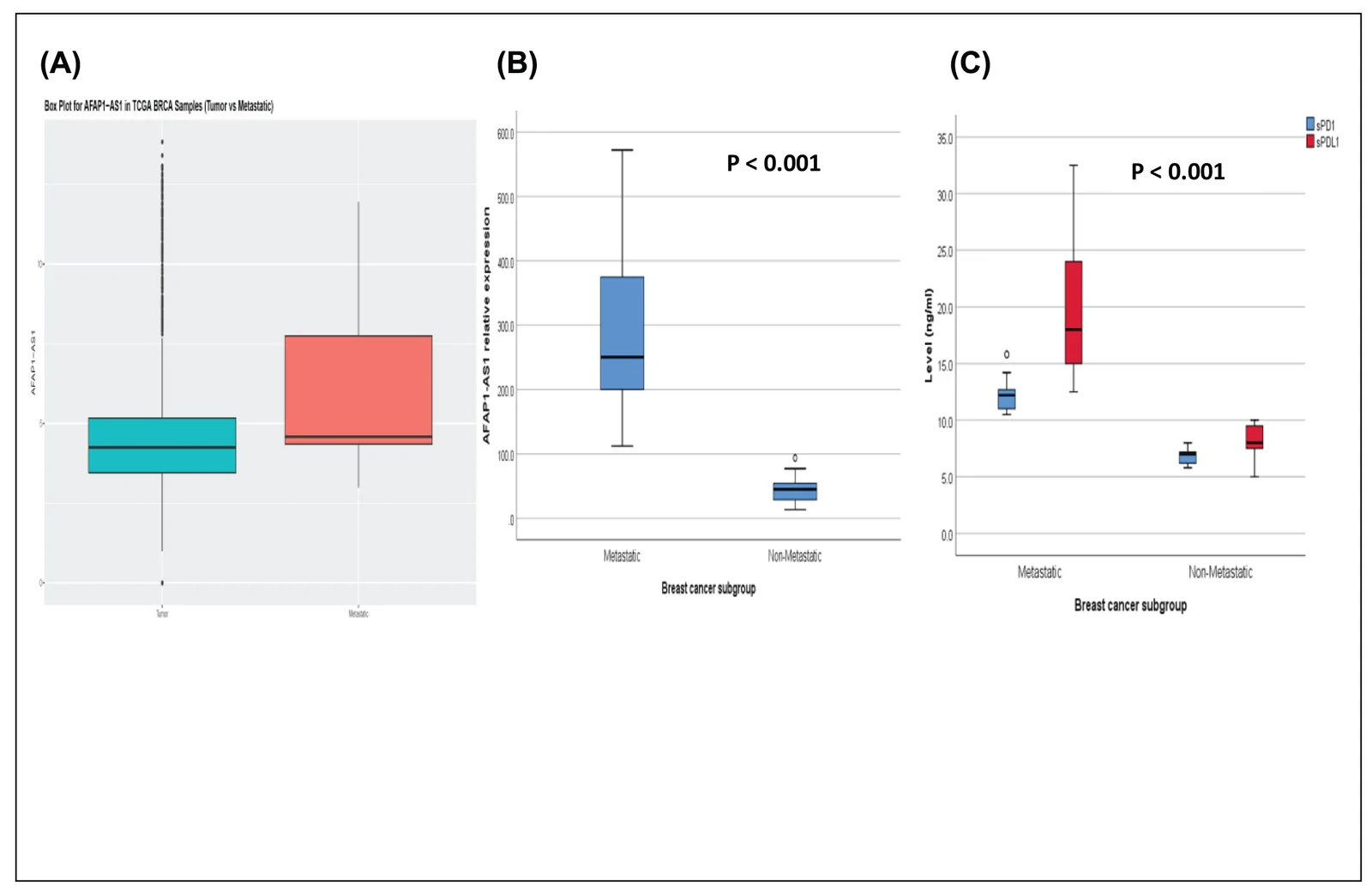
Serum AFAP1-AS1 relative expression, sPD-1, and sPD-L1 levels in metastatic and non-metastatic BC patients (**A**) Box plot for AFAP1-AS1 in TCGA-BRCA samples (tumor versus metastatic). (**B**) Serum LncRNA AFAP1-AS1 relative expression in metastatic (*n* = 45) and nonmetastatic BC (*n* = 45). (**C**) Serum sPD-1 and sPD-L1 levels in metastatic (*n* = 45) and non-metastatic BC (*n* = 45). The interquartile range in the boxplot (B,C) is represented by a box, and the median is represented by a line inside the box (Mann‒Whitney test). The minimum and maximum values are represented by error bars.

**Table 4:** Comparison of different biomarkers in metastatic and nonmetastatic BC patients

Variable	Metastatic BC (*n* = 45)		Non-metastatic BC (*n* = 45)		U-statistic	Z-statistic	*P* value^†^
	Median	IQR	Median	IQR			
**AFAP1** **-AS1 relative expression**	250.4	200.1 to 374.8	44.9	28.5 to 54.8	0.000	−8.173	<0.0001
**sPD-1 (ng/ml)**	12.2	11.0 to 12.7	7.0	6.2 to 7.2	0.000	−8.198	<0.0001
**sPD-L1 (ng/ml)**	18.0	15.0 to 24.3	8.0	7.4 to 9.5	0.000	−8.184	<0.0001

BC, Breast cancer; IQR, interquartile range.

† Mann‒Whitney test.

### Diagnostic value of serum AFAP1-AS1, sPD-1, and sPD-L1

The potential diagnostic value of AFAP1-AS1, sPD-1, and sPD-L1 in BC was assessed using ROC curve analysis, with an AUC of 1 (95% CI: 0.967–1) and 100% specificity, as demonstrated in Table 5. The cutoff values were >1.2 for AFAP1-AS1, >2.5 for sPD-1, and >2.8 for sPD-L1.

**Table 5:** ROC curve analysis for discriminating patients with BC from controls

ROC parameter	Predictor		
	AFAP1-AS1	sPD-1	sPD-L1
**AUC**	1.000	0.99	0.997
**SE**	0.000	0.00292	0.0015
**95% CI**	0.967 to 1.000	0.99 to 1.000	0.992 to 1.000
***P* value (AUC = 0.5)**	<0.0001	<0.0001	<0.0001
**Youden index J**	1.000	0.98	0.933
**Associated criterion (Cutoff)**	>1.2	>2.5	>2.8
**Sensitivity (%)**	100	98	93.3
**95% CI**	96.0–100.0	96.0–100.0	93.0–100.0
**Specificity (%)**	100	100	100
**95% CI**	83.2–100.0	83.2–100.0	83.2–100.0

ROC, receiver operating characteristic curve; AUC, area under the ROC curve; SE, standard error; 95% CI, 95% confidence interval; J-index = ([sensitivity + specificity] – 1).

### Predictive value of serum AFAP1-AS1, sPD-1, and sPD-L1

ROC curve analysis revealed the significant potential of AFAP1-AS1, sPD-1, and sPD-L1 to discriminate between metastatic and nonmetastatic BC patients, with specificities of 98.8%, 95.6%, and 97.7% and sensitivities of 98.9%, 97.8%, and 98%, respectively (Figure 3). The AUC for AFAP1-AS1 was 0.995 (95% CI: 0.984–1), with a cutoff value >93.7. For sPD-1 and sPD-L1, the AUCs were 0.991 (95% CI: 0.979–1) and 0.989 (95% CI: 0.97–1), with cutoff values >8 and >10, respectively. On the other hand, Ki-67 had an AUC of 0.82 (95% CI: 0.725–0.893), with 98% specificity, 86% sensitivity, and a cutoff value <20.

**Figure 3:**
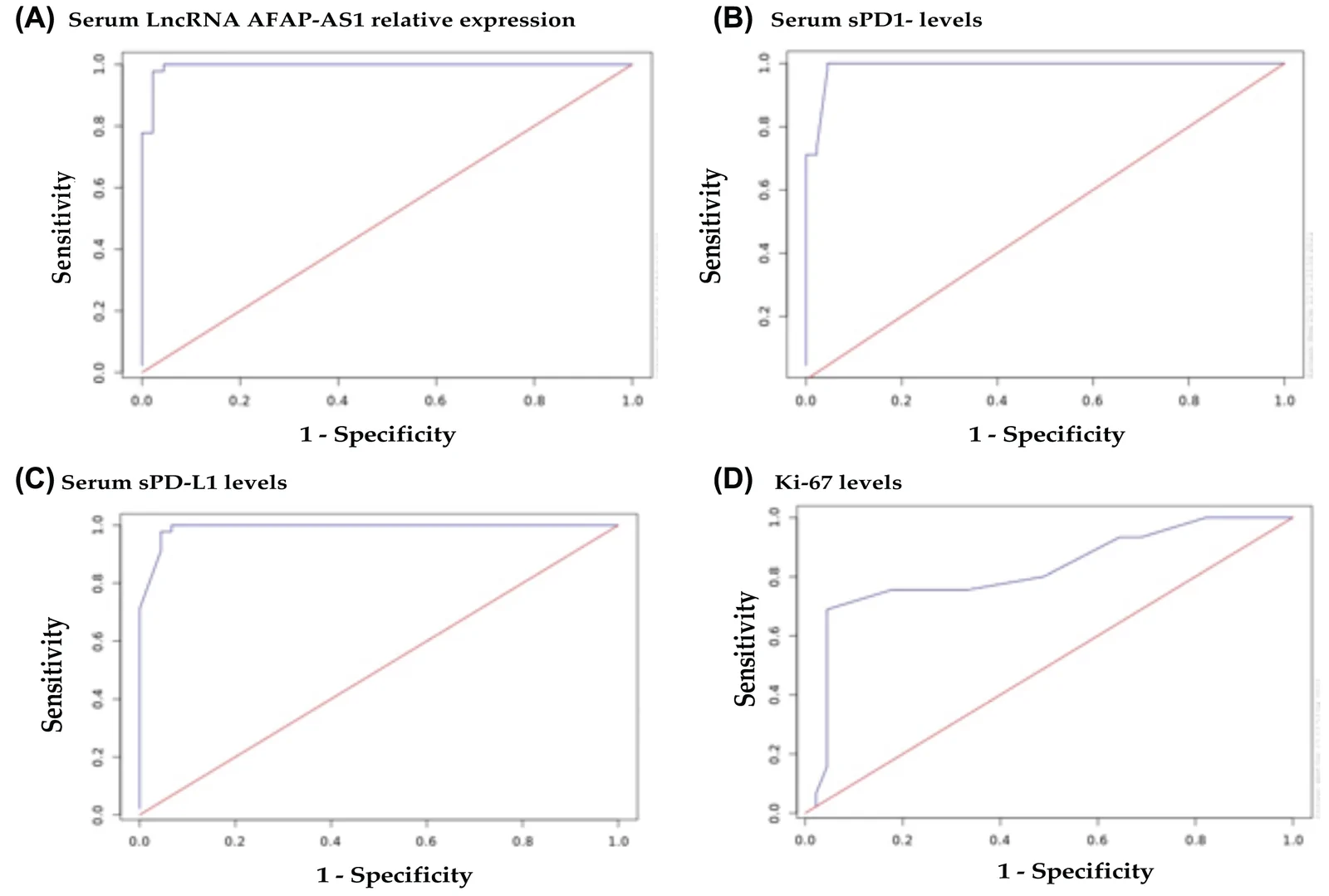
Receiver operating characteristic ROC curve for discriminating metastatic and nonmetastatic BC using (**A**) serum LncRNA serum AFAP1-AS1 relative expression, (**B**) serum sPD-1 levels, (**C**) serum sPD-L1 levels, and (**D**) Ki-67.

K-fold cross-validated ROC curves analysis was conducted to evaluate the predictive performance of each biomarker individually and in combination, with adjustment for the categorical covariate “Molecular subtype” in the case of patients with breast carcinoma. For discrimination between cases and controls, AFAP1-AS1 demonstrated perfect discriminatory ability with a cross-validated AUC of 1.000 (95% CI: 1.000–1.000). Similarly, sPDL1 showed perfect discrimination (AUC = 1.000, 95% CI: 1.000–1.000), with a cutoff of 0.800 (95% CI: 0.686–0.913). sPD1 demonstrated excellent performance with an AUC of 0.994 (95% CI: 0.975–1.000), a cutoff of 0.865 (95% CI: 0.847–0.902), and near-perfect sensitivity (98.9%) and specificity (100%). The combined model incorporating all three biomarkers also achieved perfect discrimination (AUC = 1.000, 95% CI: 1.000–1.000). These findings indicate that all three biomarkers are highly effective at distinguishing cases from normal controls (Supplementary Table S2A,B and Supplementary Figure S1).

For discrimination between cases with metastatic or non-metastatic disease as adjusted for the confounding effect of molecular subtype, AFAP1-AS1 demonstrated excellent performance with an AUC of 0.995 (95% CI: 0.988–1.000). sPD1 achieved perfect discrimination (AUC = 1.000, 95% CI: 1.000–1.000), with a cutoff of 0.760 (95% CI: 0.646–0.881). sPDL1 showed an AUC of 0.994 (95% CI: 0.972–1.000), a cutoff of 0.673 (95% CI: 0.512–0.898) yielding 95.6% sensitivity and 100% specificity. The combined model also achieved perfect discrimination (AUC = 1.000, 95% CI: 1.000–1.000). These results indicate that all three biomarkers maintain excellent predictive performance for identifying metastatic status even after accounting for molecular subtype (Supplementary Table S3A,B and Supplementary Figure S2).

For discriminating between cases with metastatic disease who have distant or contralateral-side metastasis as adjusted for the confounding effect of molecular subtype, AFAP1-AS1 showed modest discriminatory ability with an AUC of 0.569 (95% CI: 0.450–0.667) with high sensitivity (89.3%) but low specificity (48.3%), sPD1 demonstrated an AUC of 0.673 (95% CI: 0.328–0.893), though the wide CI reflects considerable variability across folds. The optimal cutoff of 0.608 (95% CI: 0.436–0.687) provided 72.7% sensitivity and 73.3% specificity. sPDL1 achieved the highest performance among individual biomarkers with an AUC of 0.707 (95% CI: 0.520–0.850). with sensitivity and 66.7% specificity. The combined model failed to improve predictive accuracy, achieving an AUC of only 0.588 (95% CI: 0.455–0.761). These findings indicate that while these biomarkers can identify the presence of metastasis, they have limited utility for distinguishing between metastatic subsites (Supplementary Table S4A,B and Supplementary Figure S3).

### Correlation analysis between the investigated biomarkers in BC patients

Pearson’s correlation analysis was performed on TCGA-BRCA samples to identify correlation of AFAP1-AS1 with PD-1 (PDCD1) and PD-L1 (CD274) in TCGA-BRCA samples and in samples with high AFAP1-AS1 levels across tumor and normal samples. Results showed that AFAP1-AS1 had a positive correlation with PD-1 (PDCD1) in TCGA-BRCA samples (r = 0.2, *P* ≤ 0.01) (Figure 4A), while it also had positive correlation with PD-L1 (CD274) (r = 0.28, *P* ≤ 0.01) (Figure 4B). Also, in TCGA-BRCA samples with high AFAP1-AS1 levels, results showed that AFAP1-AS1 had positive correlation with PD-1 (r = 0.22, *P* ≤ 0.01) (Figure 4C), as well as PD-L1 (r = 0.19, *P* ≤ 0.01) (Figure 4D). Consistent with these findings, the current study cohort showed that AFAP1-AS1 was positively correlated with sPD-1 (ρ = 0.736, *P* < .001) as well as with sPD-L1 (r = 0.746, *P* < 0.001). Additionally, there was positive association between sPD-1 and sPD-L1 (r = 0.755, *P* < 0.001) (Figure 4E). This provides evidence that AFAP1-AS1 is positively correlated with both PD-1 and PD-L1, and increased expression of AFAP1-AS1 will be a prognostic factor for increased expression of PD-1 and PD-L1 in BC patients.

**Figure 4:**
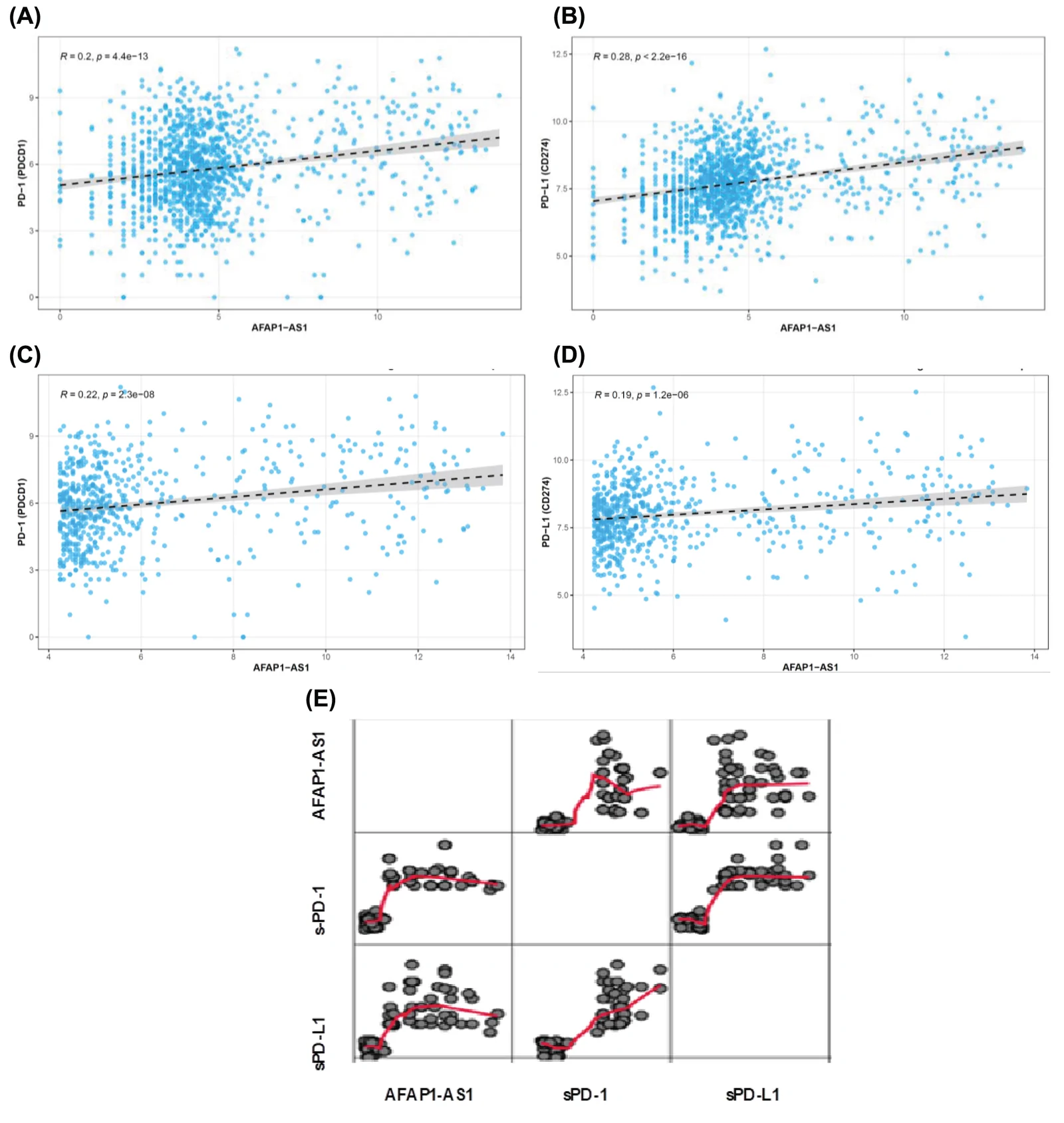
Correlation analysis for AFAP1-AS1, PD-1, and PD-L1 (**A**) Pearson’s correlation for AFAP1-AS1 and PD-1 in TCGA-BRCA samples. (**B**) Pearson’s correlation for AFAP1-AS1 and PD-L1 in TCGA-BRCA samples. (**C**) Pearson’s correlation for AFAP1-AS1 and PD-1 in TCGA-BRCA samples (high AFAP1-AS1 samples). (**D**) Pearson’s correlation for AFAP1-AS1 and PD-L1 in TCGA-BRCA samples (high AFAP1-AS1 samples). (**E**) Scatter plot matrix illustrating the correlations between various biomarkers in patients with BC. The fitted lines represent the local regression smoothing trend lines.

### Associations of the serum levels of AFAP1-AS1, sPD-1, and sPD-L1 with clinicopathological characteristics

As shown in Table 6, circulating AFAP1-AS1, sPD-1, and sPD-L1 levels were shown to be associated with positive lymph node involvement in BC patients (*P* ≤ 0.05). sPD-L1 was correlated with ER- and PR-positive tumors, whereas AFAP1-AS1 was associated with HER2-positive tumors. However, no associations were detected between any of the studied biomarkers and tumor size.

**Table 6:** Associations between the studied biomarkers and clinicopathological characteristics

Variable	AFAP1-AS1 relative expression		sPD-1 (ng/ml)		sPD-L1 (ng/ml)	
	Median (IQR)	*P* value	Median (IQR)	*P* value	Median (IQR)	P-value
**Tumor size** *T1–T2* *T3–T4*	77.2 (13.5–548.7) 133.4 (16.1–572.1)	0.095	7.4 (5.8–15.8) 11.0 (6.0–12.7)	0.88	14.0 (5.0–32.5) 15.0 (5.5–30.0)	0.81
**LN** *Negative* *Positive*	56.9 (13.5–376.0) 200.1 (14.3–572.1)	0.009^*^	7.1 (5.8–14.2) 11.0 (6.0–15.8)	0.034^*^	9.5 (5.0–32.5) 15.0 (5.0–31.0)	0.05^*^
**ER** *Negative* *Positive*	49.6 (26.0–418.8) 114.6 (13.5–572.1)	0.34	7.3 (6.0–11.2) 11.0 (5.8–15.8)	0.21	7.5 (5.0–30.0) 14.8 (5.5–32.5)	0.02^*^
**PR** *Negative* *Positive*	67.6 (27.5–418.8) 113.4 (13.5–572.1)	0.93	9.2 (6.0–11.2) 11.0 (5.8–15.8)	0.42	7.5 (5.0–30.0) 14.8 (5.5–32.5)	0.03^*^
**HER2/neu** *Negative* *Positive*	75.3 (13.5–572.1) 290.2 (221.3–548.7)	0.01^*^	11.3 (5.0–32.5) 11.0 (11.0–12.5)	0.31	11.3 (5.0–32.5) 16.0 (15.0–27.5)	0.06

T, tumor size; LN, lymph node; ER, estrogen receptor; PR, progesterone receptor; HER2, human epidermal growth factor receptor 2; IQR, interquartile range.

* Statistically significant at *P* ≤ 0.05.

### Associations of the serum levels of AFAP1-AS1, sPD-1, and sPD-L1 with molecular subtypes

Molecular subtypes were associated with different serum levels of AFAP1-AS1, sPD-1, and sPD-L1, as depicted in Table 7. Pairwise comparisons revealed that the serum AFAP1-AS1 level was significantly greater in luminal B- and HER2-positive tumors than in luminal A tumors (*P* = 0.003 and *P* = 0.021). The serum sPD-1 and sPD-L1 levels were significantly higher in the luminal B subgroup than in the luminal A subgroup (*P* < 0.001). Compared with those in TNBC patients, serum sPD-L1 levels were greater in luminal B and HER2 patients (*P* < 0.001). Ki-67 expression was greater in luminal B TNBC patients than in luminal A and TNBC patients (*P* < 0.001 and *P* = 0.006).

**Table 7:** Associations between the studied biomarkers and molecular subtypes

Variable	Molecular subtypes				
	Luminal A	Luminal B	HER2 positive	TNBC	*P* value
**AFAP1-AS1 relative expression**	48.8 (13.5–464.3)	202.3 (14.3–572.1)	290.2 (221.3–548.7)	48.8 (27.5–349.7)	<0.001*
**sPD1 (ng/ml)**	7.0 (5.8–15.8)	12.0 (6.8–15.8)	11.0 (11.0–12.5)	7.3 (6.0–11.2)	<0.001*
**sPDL1 (ng/ml)**	9.5 (5.5–25.5)	16.8 (5.6–32.5)	16.5 (15.0–27.5)	7.5 (5.0–17.5)	<0.001*
**CA 15.3 (U/ml)**	33.2 (6.7–92.6)	28.8 (10.9–92.6)	41.6 (17.0–41.6)	26.3 (15.7–38.4)	0.39
**Ki-67**	15.0 (2.0–20)	26.0 (18.0–40)	26.0 (20.0–28)	16.0 (2.0–28)	<0.001*

### *In silico* results

#### Identifying AFAP1-AS1 expression in TCGA-BRCA samples

TCGA-BRCA samples were used for calculating DEGs. Heatmaps for DEGs are shown in Figure 5. Next, volcano plots were used to identify the difference in gene expression and gene signature of DEGs in different patient categories (Figure 6A–F). Table of DEGs is provided in Supplementary Table S5.

**Figure 5:**
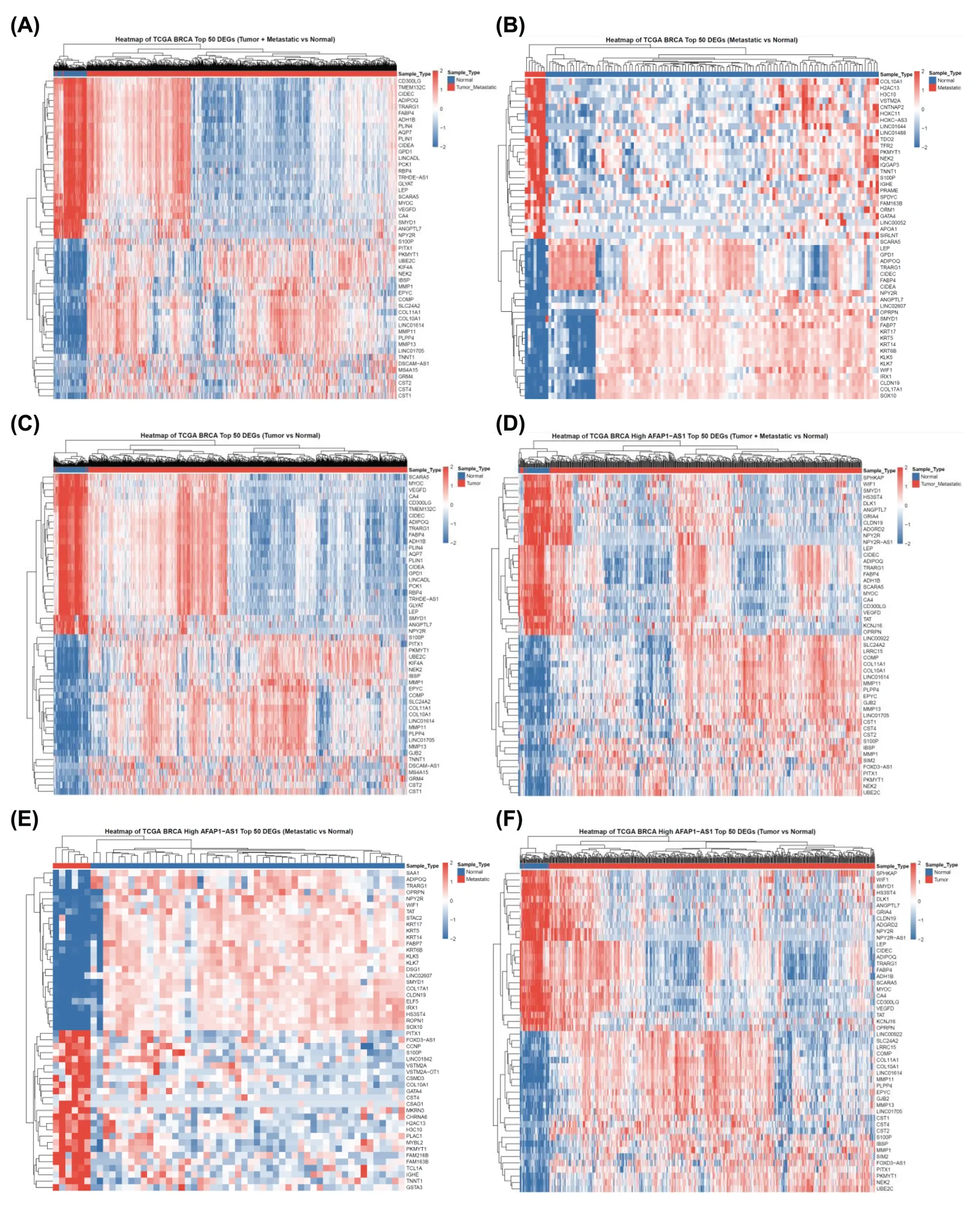
Heatmaps for DEGs in TCGA-BRCA samples (**A**) Heatmap of top 50 TCGA-BRCA DEGs (tumor + metastatic versus normal). (**B**) Heatmap of top 50 TCGA-BRCA DEGs (metastatic versus normal). (**C**) Heatmap of top 50 TCGA-BRCA DEGs (tumor versus normal). (**D**) Heatmap of top 50 TCGA-BRCA DEGs (tumor + metastatic versus normal) (high AFAP1-AS1 samples). (**E**) Heatmap for top 50 TCGA BRCA DEGs (metastatic versus normal) (high AFAP1-AS1 samples). (**F**) Heatmap of top 50 TCGA-BRCA DEGs (tumor versus normal) (high AFAP1-AS1 samples).

**Figure 6:**
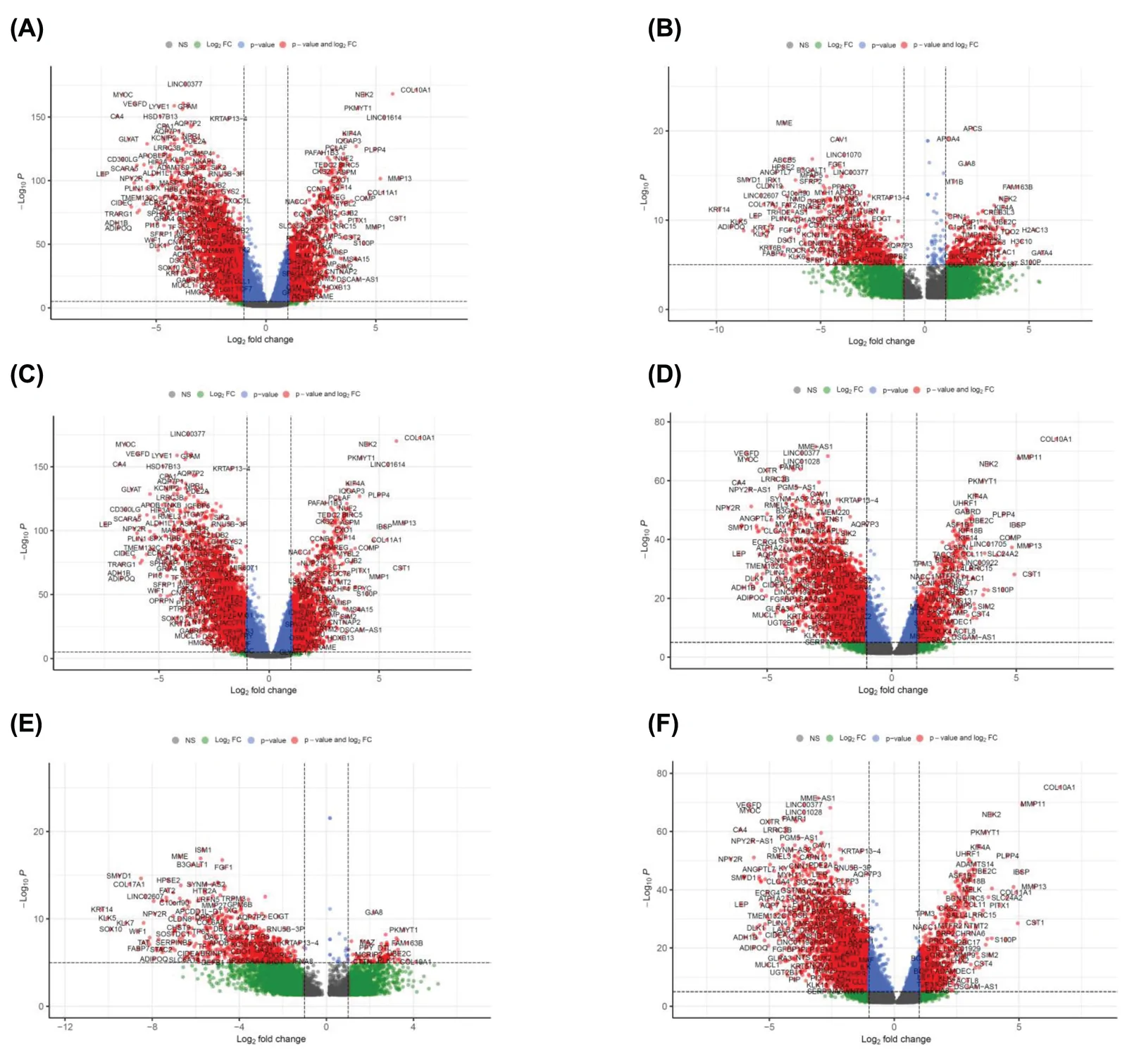
Volcano plots for AFAP1-AS1 in TCGA-BRCA samples (**A**) TCGA-BRCA DEGs (tumor + metastatic versus normal). (**B**) TCGA-BRCA DEGs (metastatic versus normal). (**C**) TCGA-BRCA DEGs (tumor versus normal). (**D**) TCGA-BRCA DEGs (tumor + metastatic versus normal) (high AFAP1-AS1 samples). (**E**) TCGA-BRCA DEGs (metastatic versus normal) (high AFAP1-AS1 samples). (**F**) TCGA-BRCA DEGs (tumor versus normal) (high AFAP1-AS1 samples).

#### GSEA provides distinct molecular attributes for AFAP1-AS1 in TCGA-BRCA samples

Based on top 50 DEGs, GSEA was performed using GO database (https://geneontology.org/) to annotate gene function networks in all studied conditions (TCGA-BRCA samples and high-AFAP1-AS1 TCGA-BRCA samples). GSEA was specified to include attributes related to biological processes and cellular components. Results showed that the top gene attributes for biological processes in TCGA-BRCA samples include extracellular matrix disassembly, proteolysis, and extracellular matrix organization as activated processes through genes including MMP13, COL11A1, IBSP, and MMP11, while vesicle-mediated transport, cellular response to stimulus, and intracellular transport were suppressed functions through FABP4, GPD1, VEGFD, and SCARA5 (Figure 7A,B). In case of TCGA-BRCA samples with high AFAP1-AS1 levels, GSEA found that cellular component disassembly, extracellular structure organization, and external encapsulating structure organization were activated via CST1, CST2, CST4, and UBE2C, while protein tyrosine kinase metabolic process, organic acid metabolic process, and MAPK cascade were suppressed via ADH1B, CA4, and ADIPOQ (Figure 7C,D). This proves that gene sets of activated or suppressed genes in TCGA-BRCA samples and in samples with high AFAP1-AS1 levels show distinct patterns for tumor progression. All figures for TCGA BRCA GO cellular components are provided in Supplementary Figure S4.

**Figure 7:**
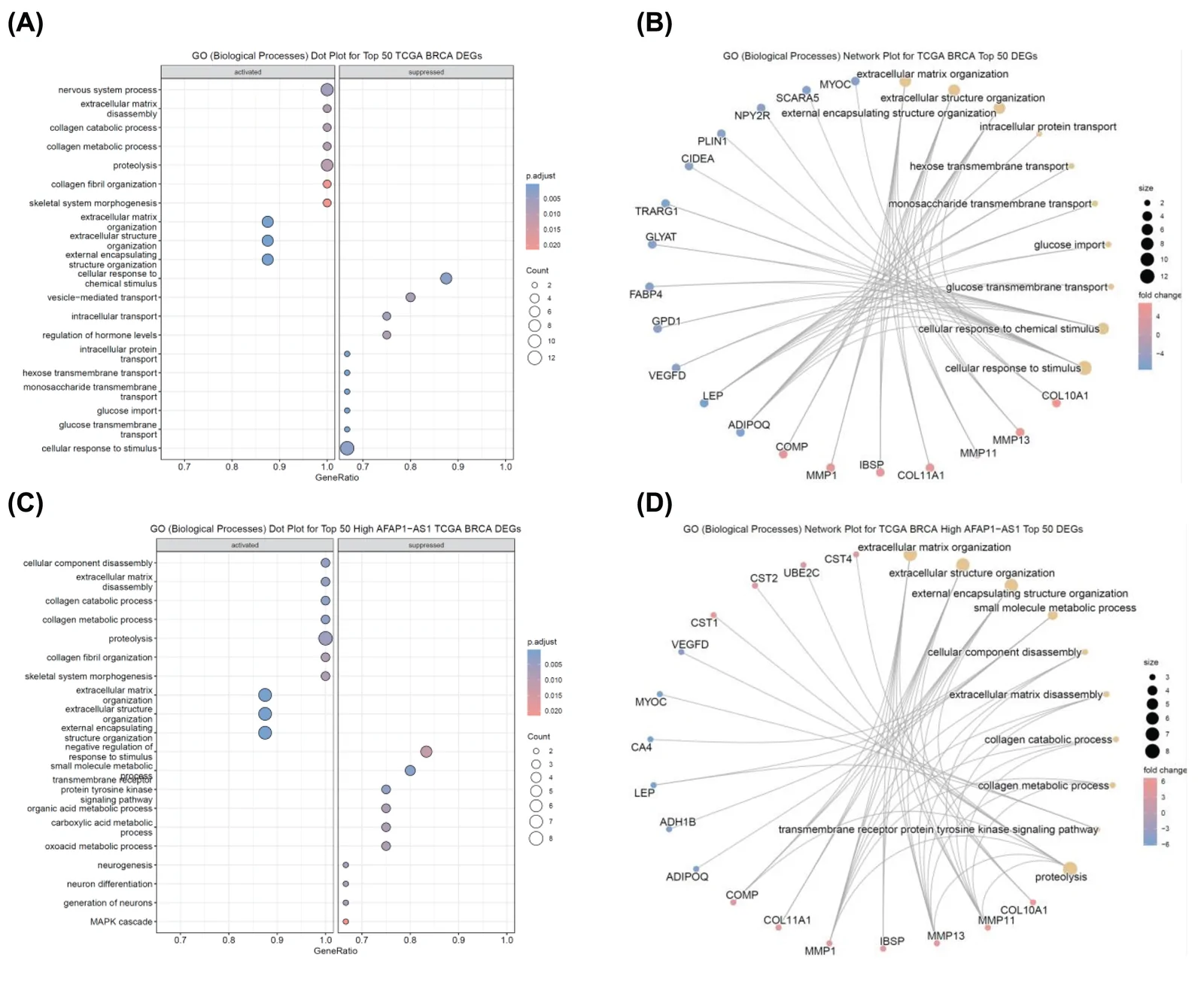
Gene set enrichment analysis for AFAP1-AS1 in TCGA-BRCA samples (**A**) GO (biological processes) dot plot for top 50 TCGA-BRCA DEGs. (**B**) GO (biological processes) network for top 50 TCGA-BRCA DEGs. (**C**) GO (biological processes) dot plot for top 50 TCGA-BRCA DEGs (high AFAP1-AS1 samples). (**D**) GO (biological processes) network for top 50 TCGA-BRCA DEGs (high AFAP1-AS1 samples).

#### Survival analysis

Survival analysis of TCGA BRCA data was conducted. Samples were categorized into high/low AFAP1-AS1 levels based on median expression level of AFAP1-AS1 in each studied category. High AFAP1-AS1 expression was associated with shorter PFS (*P* = 0.03) (Figure 8A). In addition, worse OS was observed in cases with elevated AFAP1-AS1 expression (Figure 8B). Thus, KM plot results show that high AFAP1-AS1 expression has potential prognostic value for survival of BC patients. TCGA BRCA survival analysis data results are provided in Supplementary Tables S6 and S7.

**Figure 8:**
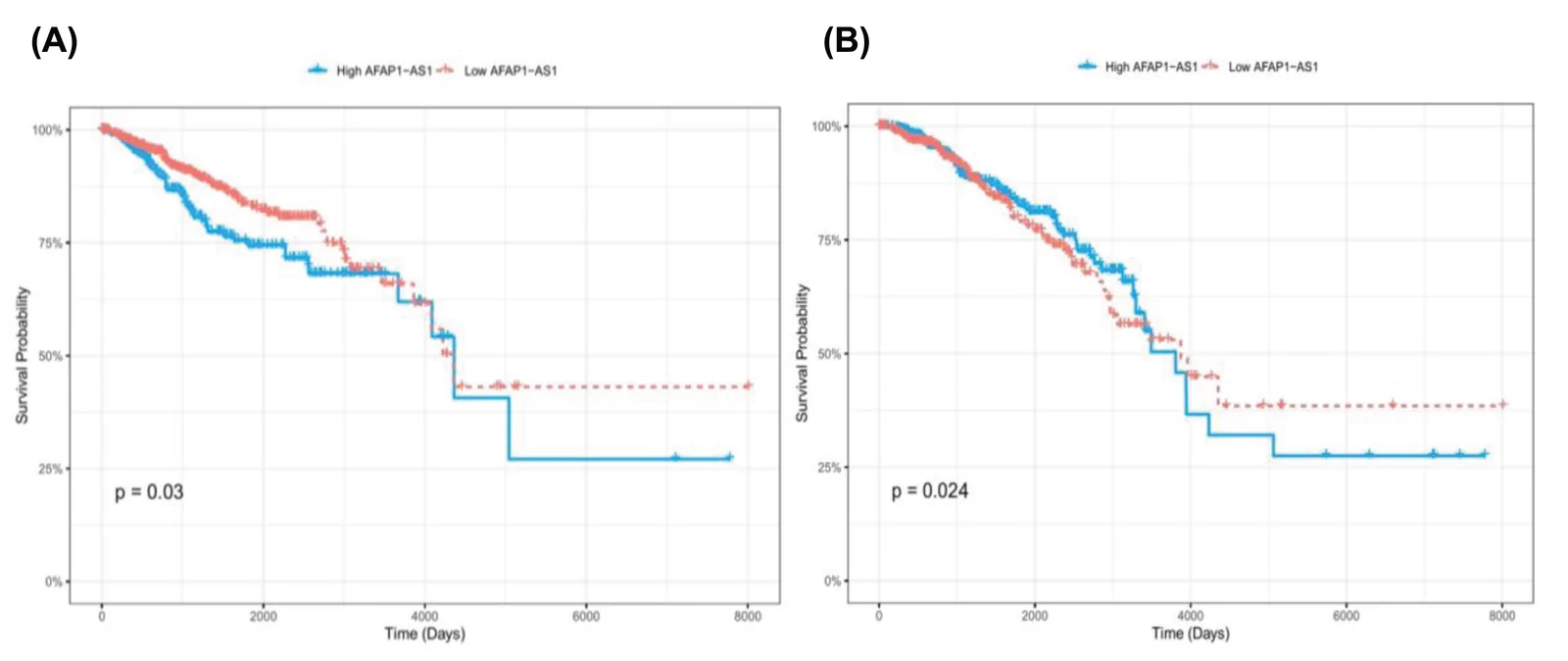
Survival analysis for AFAP1-AS1 in TCGA-BRCA samples (**A**) Kaplan–Meier PFS plot for TCGA-BRCA samples (high AFAP1-AS1 versus low AFAP1-AS1). (**B**) Kaplan–Meier OS plot for TCGA-BRCA samples (high AFAP1-AS1 versus low AFAP1-AS1).

#### Prediction of AFAP1-AS1 and PD-1 and PD-L1 interaction probabilities

The interaction probabilities between AFAP1-AS1 and PD-1 and PD-L1 were predicted via RPISeq. The interaction probabilities generated by RPISeq range from 0 to 1, where values greater than 0.5 are considered positive predictions, indicating a likely interaction between the RNA and protein. Predictions from both the RF and SVM models were recorded. Both the RF and SVM models predicted interaction probabilities greater than 0.5 for the AFAP1-AS1–PD-1 pair (0.70 and 0.81, respectively), indicating a strong likelihood of interaction. For the AFAP1-AS1–PD-L1 pair, the RF (0.65) and SVM (0.52) predictions also exceeded the 0.5 threshold, suggesting a potential interaction.

#### Proteins that bind to the lncRNA AFAP1-AS1 via the RNAct database

Proteins that bind to the *lncRNA AFAP1-AS1* were examined via the RNAct database https://rnact.crg.eu. These proteins were found to be related to BC progression and metastasis (Supplementary Table S8).

#### Regulatory interactions of the lncRNA AFAP1-AS1 with potential target miRNAs

The comprehensive web-based enrichment analysis tool Enrichr (https://maayanlab.cloud/Enrichr/) was utilized. The analysis yielded a ranked list of miRNAs on the basis of statistical significance, including p values and adjusted q values (Benjamini‒Hochberg correction). From the results, we selected the top 10 miRNAs with the most significant enrichment, on the basis of the lowest p values and q values, for further functional analysis and visualization (Figure 9). These miRNAs are considered key regulatory candidates in the lncRNA-mediated posttranscriptional regulatory network. Appyter (https://appyters.maayanlab.cloud/Enrichment_Analysis_Visualizer/7572b5fcd9fa432a817bf34a3262b0843111ad83/), was used to create a variety of visualizations for enrichment analysis results for our resulting Enrichr library.

**Figure 9:**
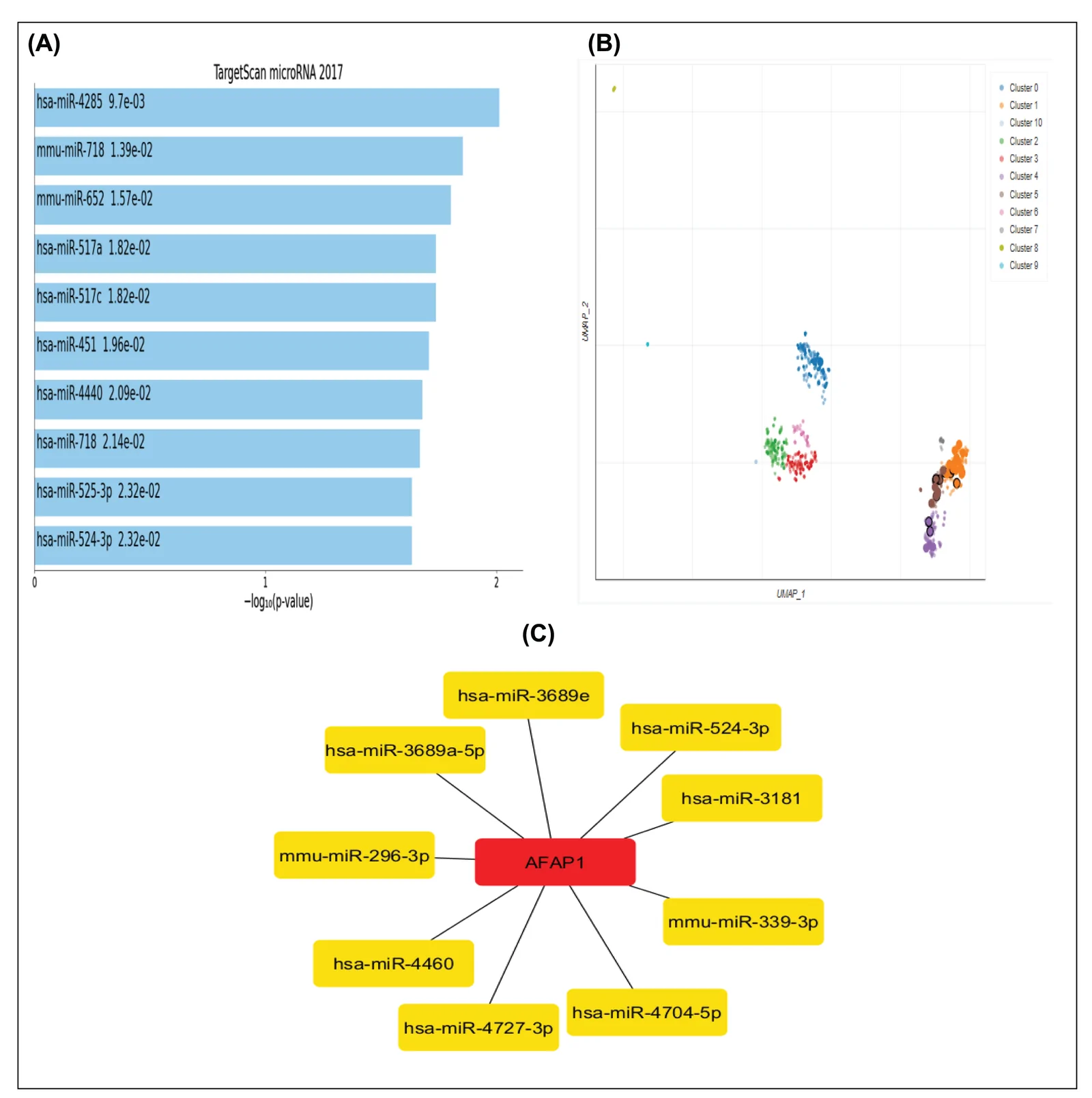
Regulatory interactions of the lncRNA AFAP1-AS1 with potential target miRNAs (**A**) Bar chart of the top enriched terms from the TargetScan_microRNA_2017 gene set library. The top 10 enriched terms for the input gene set are displayed on the basis of the −log_10_(p value), with the actual p value shown next to each term. The term at the top has the most significant overlap with the input query gene set. (**B**) Scatterplot of all terms in the TargetScan_microRNA_2017 gene set library. Each point represents a term in the library. Term frequency-inverse document frequency (TF-IDF) values were computed for the gene set corresponding to each term, and UMAP was applied to the resulting values. The terms are plotted on the basis of the first two UMAP dimensions. Generally, terms with more similar gene sets are positioned closer together. The terms are colored according to the automatically identified clusters computed with the Leiden algorithm applied to the TF-IDF values. The darker and larger the point is, the more significantly enriched the term. (**C**) Network analysis was conducted via the cytoHubba plugin within Cytoscape, highlighting the top 10 nodes ranked by MCC.

#### Functional enrichment analysis via the KEGG pathway

The pathways associated with PD-1 and PDL-1 identified from the KEGG database https://www.genome.jp/kegg/kegg1.html are shown in Figure 10. The KEGG pathways targeted by PD-L1 include the PI3K-Akt signaling pathway, the HIF-1 signaling pathway, the MAPK signaling pathway, and the Toll-like receptor signaling pathway. The T-cell receptor signaling pathway, calcium signaling pathway, MAPK signaling pathway, and PI3K-Akt signaling pathway are the targeted KEGG pathways for PD-1.

**Figure 10:**
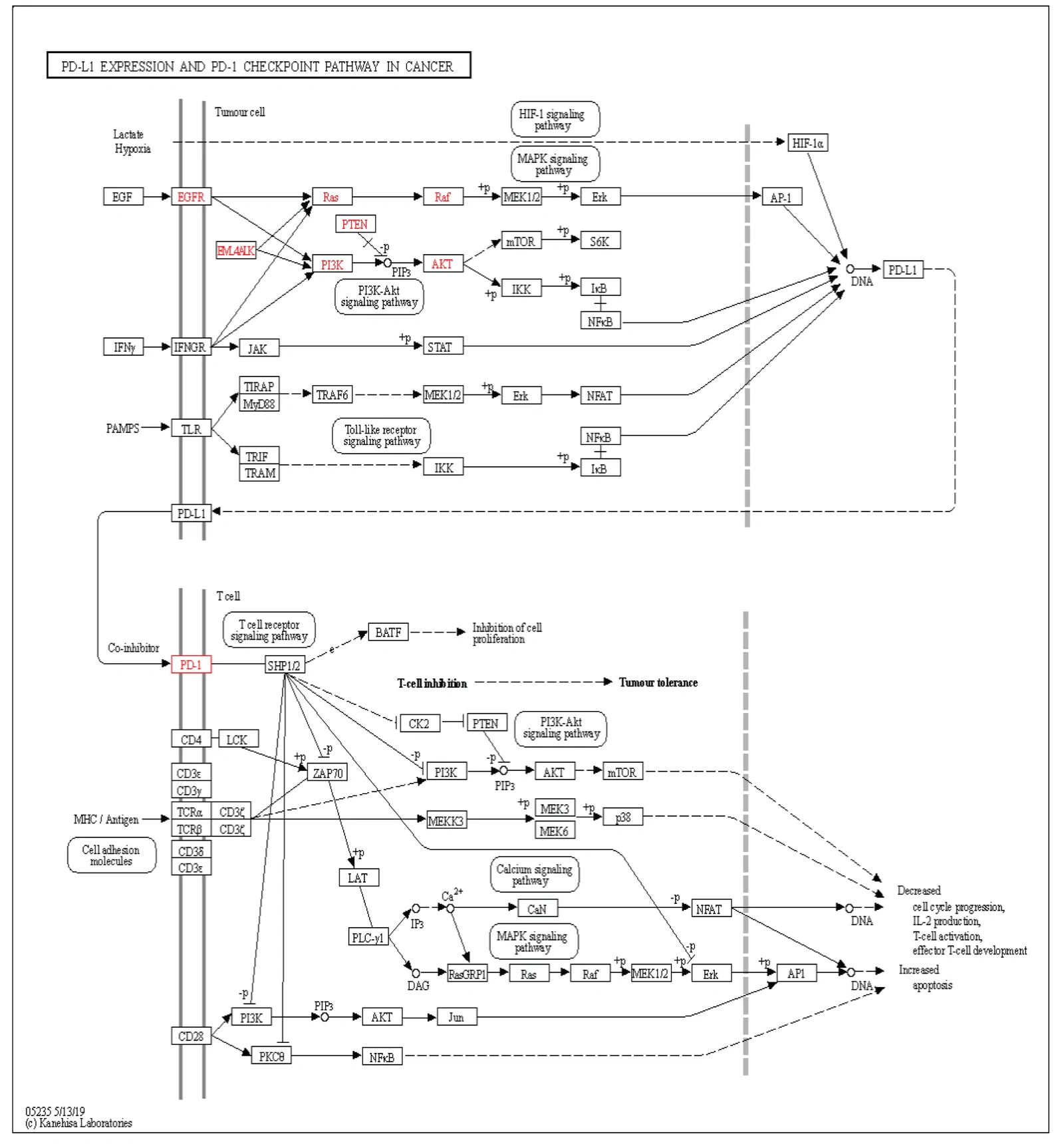
Pathways associated with PD-1 and PDL-1 identified from the KEGG database

#### Functional protein association network

PD-1 and PD-L1 signaling pathway data were retrieved from the Reactome database https://reactome.org (Figure 11). The protein‒protein interactions of PD-1 and PD-L1 and the functional protein association network from the STRING database https://string-db.org are depicted in Figure 12A,B. In addition, Figure 12C,D shows the interactions of PD-1 and PD-L1 with other genes from the Pathway commons database https://ngdc.cncb.ac.cn/databasecommons. The IntAct Network for PD-1 and PD-L1 is shown in Figure 12E,F.

**Figure 11:**
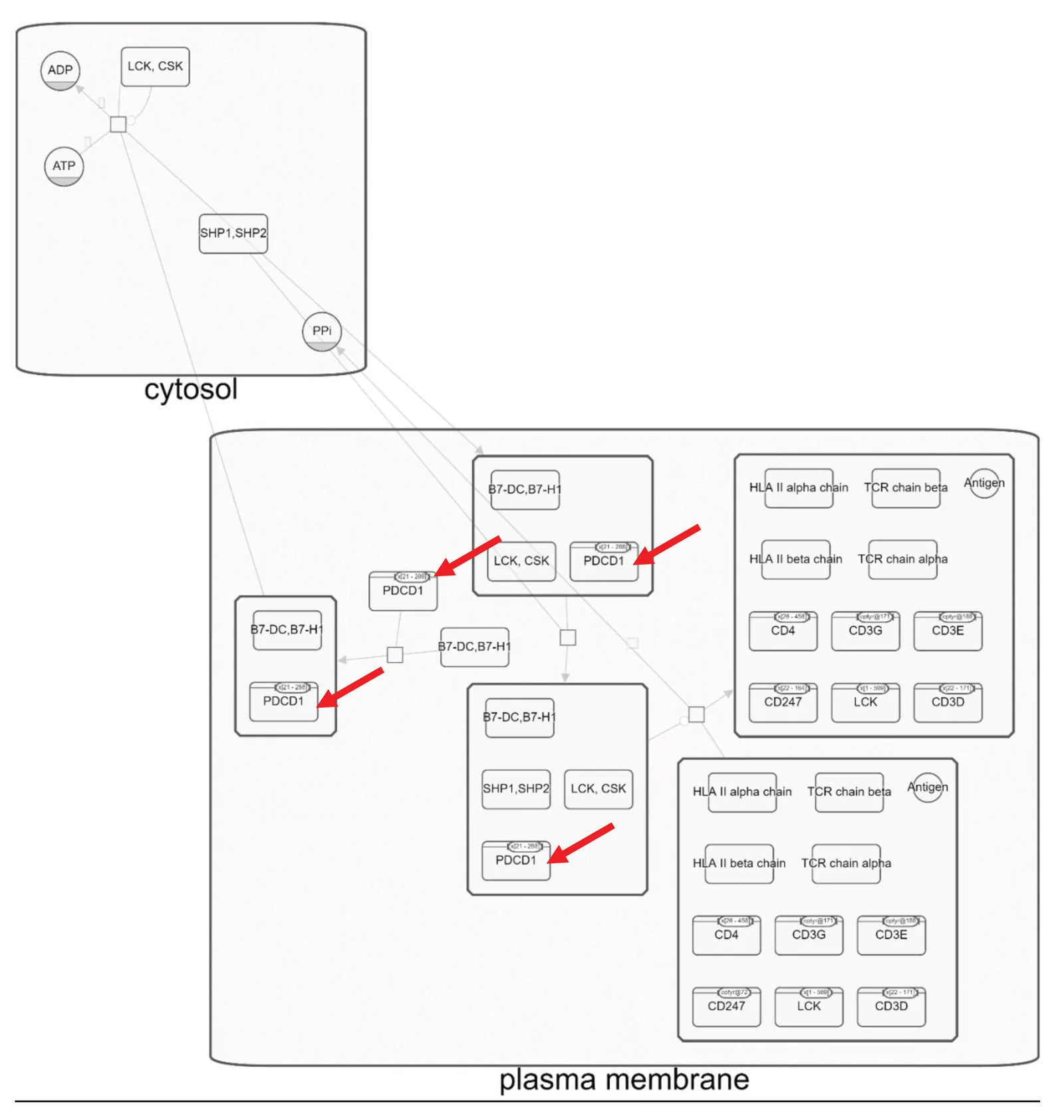
PD-1 signaling pathway from the Reactome database (involving PD-1 and PDL-1)

**Figure 12:**
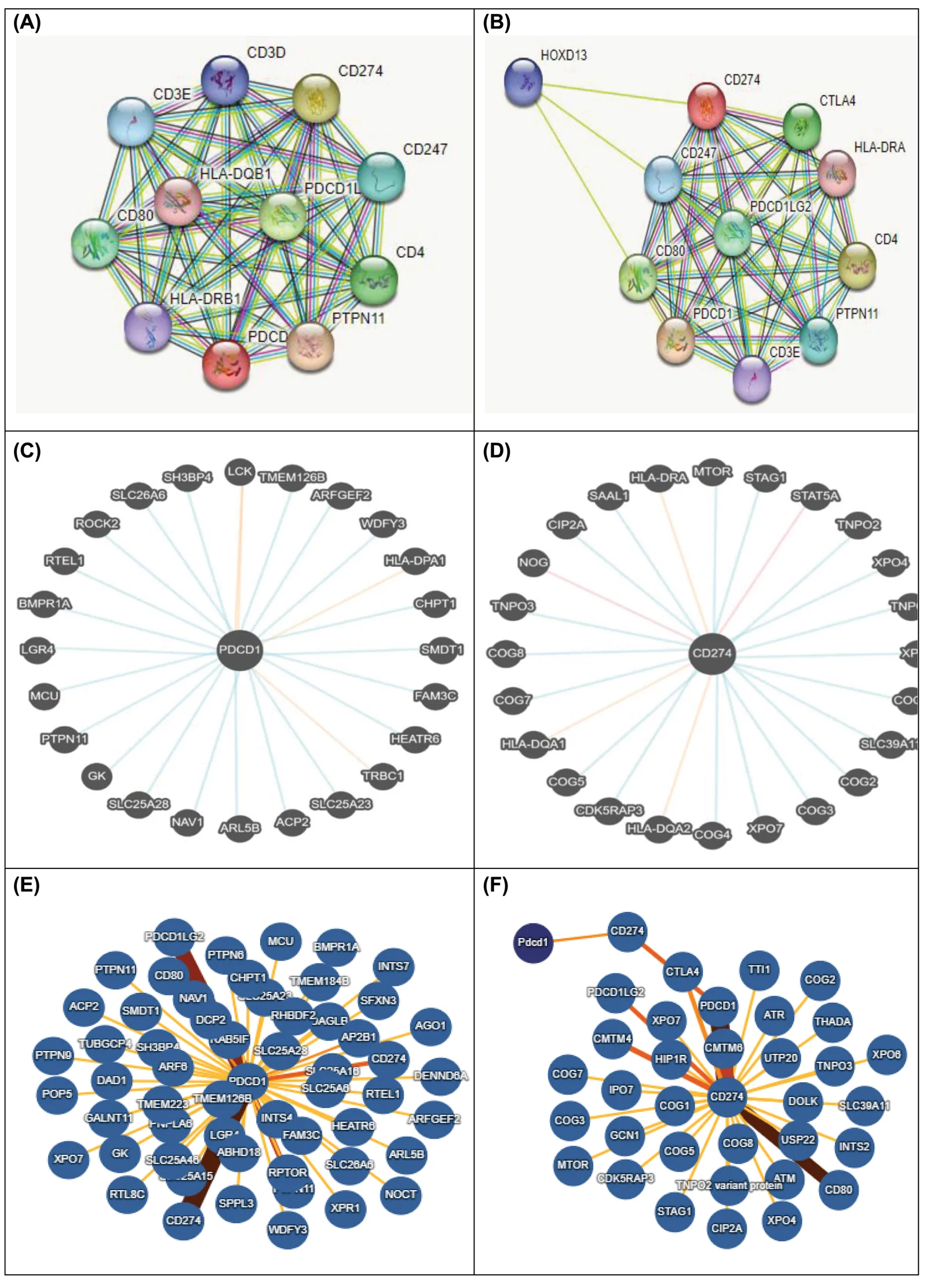
Protein‒protein interactions of PD-1 and the PD-L1 functional protein association network Protein‒protein interactions of (A) PD-1, and (B) PDL-1 from the STRING database. Interactions of (C) PD-1 and (D) PD-L1 with other genes from the pathway commons database. IntAct Network for (E) PD-1 and (F) PD-L1.

## Discussion

Liquid biopsy has attracted considerable attention in immunotherapy for the study of diagnostic and predictive biomarkers or for monitoring the response to therapy [46]. The present study aimed to investigate the potential significance of the lncRNAs AFAP1-AS1, sPD-1, and sPD-L1 as diagnostic biomarkers for BC patients and their prognostic value, in addition to identifying their regulatory pathways.

LncRNAs are involved in multiple biological processes, including the modulation of the immune response [47]. Several lncRNAs are aberrantly expressed in a variety of cancers and are implicated in numerous stages of tumorigenesis, including development and metastasis [48]. The potential utility of lncRNAs, including NORAD [49], HISLA [50], TROJAN [51], H19 [52], SNHG1 [53], and DDX11-AS1 [54], has recently been investigated as biomarkers in BC. Emerging evidence also shows that lncRNAs play pivotal roles in cancer immunity. For example, the lncRNA SNHG1 was shown to regulate the differentiation of regulatory T cells and impact immune escape in BC [55]. Additionally, the close association of lncRNAs with BC metastasis has been revealed in many studies [56,57].

In the present study, serum AFAP1-AS1 expression was significantly higher in BC patients than in control subjects. Moreover, expression levels were significantly higher in metastatic than in nonmetastatic patients, similar to the findings of previous studies [58], and were found to be associated with positive lymoh node involvement. Dianatpour *et al.* previously reported the up-regulation of AFAP1-AS1 expression in BC tumor tissue samples compared with adjacent noncancerous tissues and BC cell lines, suggesting its involvement in BC pathogenesis [59]. Zhang *et al.* also revealed that up-regulated AFAP1-AS1 expression promotes cell proliferation and invasion in TNBC cells *in vitro* in addition to stimulating tumor growth in nude mice [38]. Moreover, Ma *et al.* reported that AFAP1-AS1 expression in BC tissue was associated with malignancy status and poor prognosis, while the knockdown of AFAP1-AS1 resulted in the suppression of proliferation and the induction of apoptosis in BC cells [60]. In addition, studies have demonstrated that AFAP1-AS1 can modulate the progression of BC cells and affect tumorigenesis in mice by acting as a miR-2110 sponge, resulting in the regulation of Sp1 expression [61]. Cai *et al.* revealed that interference with AFAP1-AS1 inhibited the progression of BC by modulating SEPT2 by competitively binding with miR-497-5p [62]. Recently, Li *et al.* reported that AFAP1-AS1, which is activated by the transcription factor SP1, can activate the mTOR pathway, consequently facilitating the progression of TNBC, suggesting that AFAP1-AS1 might be a therapeutic target for TNBC [63]. The expression of the lncRNA AFAP1-AS1 was found to be elevated in numerous cancers and was associated with advanced stages and larger tumor sizes in patients with colorectal cancer [39], esophageal squamous carcinoma [64], and lung cancer [65]. The up-regulation of AFAP1-AS1 has also been reported to promote invasion and metastasis in nasopharyngeal carcinoma (NPC) [66].

The PD-1/PD-L1 axis has been implicated in cancer immune escape and tumor progression [21]. Recently, PD-L1 was shown to be overexpressed in many types of tumors, including ovarian cancer, breast adenocarcinoma, lung squamous cell carcinoma, melanoma, and colon adenocarcinoma [12,67]. PD-L1 expression was found to be associated with epithelial-mesenchymal transition in human BC stem cells and hence plays a vital role in the incidence and progression of BC [68,69]. IHC studies revealed that PD-L1 expression in BC was associated with poor survival outcomes [70,71]. A genomic analysis of the mRNA expression of different immune regulatory molecules in BC patients demonstrated a significant correlation between the PD-L1 gene and the degree of disease progression [70,72].

Several studies have demonstrated increased levels of sPD-1 or sPD-L1 in numerous cancers, including renal cell carcinoma [29], hepatocellular carcinoma [73], gastric cancer [74], lung cancer [30], esophageal cancer [75], and pancreatic cancer [76]. Similarly, the current study revealed that serum sPD-1 and sPD-L1 levels were relatively elevated in BC patients compared with healthy controls, indicating their potential efficacy as diagnostic biomarkers. Moreover, their levels were significantly elevated in metastatic BC patients compared with nonmetastatic BC patients and were positively associated with lymph node involvement. This finding is in agreement with recent studies showing that high plasma levels of sPD-1 and sPD-L1 are associated with progressive disease [14,28,77]. sPD-L1 concentrations were shown to predict metastasis in patients with soft tissue sarcoma [78]. Moreover, high pretreatment sPD-L1 levels are associated with progressive disease and are an independent prognostic factor for lower PFS and OS in numerous cancer patients [79]. Soluble PD-L1 is a predictive and prognostic biomarker in advanced cancer patients who receive immune checkpoint blockade treatment. Recently, elevated sPD-L1 was shown to be significantly associated with shorter PFS and OS in metastatic BC patients [80,81]. In addition, Li *et al.* demonstrated that sPD-1 and sPD-L1 could be used as noninvasive predictive biomarkers for the response to chemotherapy in TNBC patients [82]. In addition, soluble PD-L1 was reported to be a potential dynamic predictive biomarker for immunotherapy in patients with proficient mismatch repair-related colorectal cancer [83].

Our results also revealed a significant positive correlation between the serum levels of sPD-1 and sPD-L1, suggesting a communal source and that both molecules are coregulated, which is consistent with the findings of previous studies [76,82]. An increase in both sPD-1 and sPD-L1 indicates that they may exert regulatory mechanisms to counter each other’s role in a way similar to membrane-bound up-regulation [14]. Functional enrichment analysis via targeted KEGG pathway analysis revealed that PD-1 and PD-L1 are involved in several signaling pathways implicated in BC progression, including the PI3K-Akt, HIF-1, MAPK, Toll-like receptor, T cell, and calcium signaling pathways. In addition, PD-1 and PD-L1 interact with several proteins related to immune activation and tumor invasion.

AFAP1-AS1 expression was found to be positively correlated with sPD-1 and sPD-L1 levels. These findings were further supported by TCGA BRCA dataset analysis, suggesting a regulatory relationship between the lncRNA AFAP1-AS1 and PD-1/PD-L1. In addition, RPISeq analysis predicted that AFAP1-AS1 could physically interact with both PD-1 and PD-L1, with interaction probabilities exceeding the 0.5 positivity threshold across both machine learning models. These findings suggest that AFAP1-AS1 may participate in immune checkpoint regulation by directly interacting with the PD-1 and PD-L1 proteins. Additionally, the RNAct database revealed potential interactions between AFAP1-AS1 and other proteins related to BC progression and metastasis, including LIN28B, WDR43, UPF1, FUS, AKAP8L, SAFB2, IGF2BP2, and DDX3X. While the *in silico* analysis suggests a putative regulatory association between AFAP1-AS1 and the PD-1/PD-L1 axis, experimental validation would confirm the proposed mechanistic link.

AFAP1-AS1 is thought to be involved in the regulation of PD-1 expression through several mechanisms. First, AFAP1-AS1 can act as a competing endogenous RNA (ceRNA) for PD-1 and is also recognized as a pseudogene [66,84,85]. Alternatively, AFAP1-AS1 may regulate PD-1 transcription through binding to certain transcriptional complexes. AFAP1-AS1 was shown to be involved in the epigenetic modification of PD-1 through direct binding with the PDCD1 gene [66,86,87]. A possible RNA–RNA interaction was found between AFAP1-AS1 and phosphoprotein associated with glycosphingolipid-enriched microdomains 1 (PAG), a downstream effector of PD-1 that contributes to the inhibitory function of PD-1 in T cells. Therefore, a possible mechanism of action of AFAP-AS1 could be related to the PD-1/PAG axis [88]. Moreover, coexpression of AFAP1-AS1 and PD1 in biopsies of NPC patients was associated with a poor prognosis [47,89].

In the present study, analysis of TCGA BRCA data identified 22 831 DEGs, including AFAP1-AS1, across tumor samples compared with control. AFAP1-AS1 was statistically significant and up-regulated in BC tumor samples compared with control (FC = 4.52), which is concurrent result from other BC studies [38]. Samples were classified into high/low AFAP1-AS1 based on median expression level. Li *et al.* found that AFAP1-AS1 GO in BC samples is attributed to extracellular matrix and extracellular matrix organization [63], which were also found in our study in GO analysis of TCGA-BRCA samples with linkage to MMP13, COL11A1, IBSP, and MMP11 up-regulated genes.

In the present study, sPD-L1 was associated with ER- and PR-positive tumors, whereas AFAP1-AS1 was associated with HER2-positive tumors. Luminal A breast tumors are known to have a low proliferation rate, whereas luminal B tumors are more aggressive and have a significantly worse prognosis [90]. A previous study revealed that PD-1 had the lowest expression in the luminal A subtype [91]. Notably, the results of the present study revealed that the luminal B molecular subtype presented significantly higher levels of AFAP1-AS1, sPD-1, and sPD-L1 than did the luminal A subtype. These results indicate that the serum AFAP1-AS1, sPD-1, and sPD-L1 levels may vary among different molecular subtypes, which warrants further investigation in larger cohorts.

To assess the robustness of our findings and the lack of external validation in independent cohorts, we performed a k-fold (5-fold) cross-validated ROC analysis with adjustment for the categorical covariate “Molecular subtype”. All three biomarkers demonstrated excellent ability to distinguish cases from normal controls, and to discriminate between metastatic and non-metastatic disease after adjustment for molecular subtype. However, distinguishing between distant and contralateral metastasis among patients’ discriminatory performance was substantially reduced. These results indicate that AFAP1-AS1, sPD1, and sPDL1 are robust predictors of both disease status and metastatic presence, with predictive performance independent of molecular subtype, but they have limited utility for differentiating site of metastasis, suggesting that site of metastasis is governed by biological mechanisms distinct from those driving metastasis *per se*.

Moreover, TCGA BRCA dataset survival analysis was performed to identify the prognostic impact of AFAP1-AS1 in BC settings. Results showed that high AFAP1-AS1 in TCGA BRCA has poor PFS as well as poor OS compared with low AFAP1-AS1 BC samples (*P* = 0.024, *P* = 0.03), which is also evident in other BC studies in which low AFAP1-AS1 was also linked to enhanced survival of BC patients [58,61]. These findings provide robust *in silico* validation of the independent prognostic value of AFAP1-AS1 in BC, drawing upon a dataset with substantially greater sample size and follow-up maturity than our clinical cohort. Nevertheless, there are cohort-level compositional differences between public databases and clinical study populations, including baseline characteristics, where public datasets incorporate heterogeneous cohort spanning multiple BC molecular subtypes, treatment regimens, and staging categories. In addition, public datasets include tissue/tumor-derived lncRNA expression derived from bulk RNA sequencing datasets, while liquid biopsy-derived measurement reflects the lncRNA shed into peripheral blood, which are distinct biological entities. Moreover, datasets interrogated by public platforms display median follow-up durations and event (death/progression) maturity that may be different from single-institution clinical cohorts.

Collectively, our data support circulating AFAP1-AS1 as a candidate prognostic biomarker for BC metastasis, warranting further validation in prospective multicenter cohorts with mature clinical follow-up. Given the significant positive associations observed in our study and supported by the *in silico* analysis between AFAP1-AS1 and both sPD-1 and sPD-L1, as well as between sPD-1 and sPD-L1, it suggests that these markers collectively reflect a coordinated immune evasion axis, and so composite multi-marker survival models incorporating AFAP1-AS1 together with sPD-1 and/or sPD-L1 may demonstrate substantially stronger significant prognostic associations. Although internal cross-validation analysis supported the robustness of our findings, external validation in larger, independent, and multi-center cohorts is necessary before these markers can be considered for clinical application. Future studies incorporating prospectively collected, larger patient cohorts are warranted.

## Conclusions

Liquid biopsy has emerged as a breakthrough in medical oncology, paving the way for the era of precision medicine. The present study highlights the potential clinical value of serum lncRNA AFAP1-AS1, sPD-1, and sPD-L1 as non-invasive biomarkers for the early detection of BC and the prediction of metastasis. Circulating AFAP1-AS1 demonstrated significant correlations with features of BC aggressiveness and metastatic potential in our clinical cohort, while *in silico* TCGA-BRCA analysis provided independent supporting evidence for its prognostic value with respect to both OS and PFS. Prospective longitudinal validation of the composite prognostic model comprising circulating AFAP1-AS1, sPD-1, and sPD-L1 in an extended cohort with mature follow-up is warranted. These biomarkers may also represent potential therapeutic targets that can be employed to stimulate antitumor immunity and prevent metastasis. There is, nonetheless, a lengthy path to decipher the intricacies of tumor–immune cross-talk and immune escape strategies.

## Supplementary Material

Supplementary Figures S1-S4

Supplementary Tables S1-S8

## Data Availability

All the data generated or analyzed during the present study are included in this published article.

## Abbreviations

AFAP1-AS1: Actin filament-associated protein 1-antisense RNA 1
AJCC: American Joint Committee on Cancer
ASCO/CAP: American Society of Clinical Oncology/College of American Pathologists
AUC: Area under the curve
BC: Breast cancer
BH: Benjamini–Hochberg
BIRADS: Breast imaging-reporting and data system
BRCA: Breast cancer gene
ceRNA: competing endogenous RNAs
DEGs: Differentially expressed genes
ELISA: Enzyme-linked immunosorbent assay
ER: Estrogen Receptor
FPR: False positive rates
GDV: Genome Data Viewer
GO: Gene ontology
GSEA: Gene set enrichment analysis
HER2: Human epidermal growth factor receptor 2
HGNC: HUGO Gene Nomenclature Committee
IQR: Interquartile range
KM: Kaplan–Meier
KEGG: Kyoto Encyclopedia of Genes and Genomes
LncRNA: Long non-coding RNA
MCC: Maximal Clique Centrality
NCBI: National Center for Biotechnology Information
NK cells: Natural killer cells
NPC: Nasopharyngeal carcinoma
OS: Overall survival
PAG: Phosphoprotein associated with glycosphingolipid-enriched microdomains 1
PFS: Progression-free survival
PPI: Protein-protein interaction
PR: Progesterone receptor
qRT‒PCR: Quantitative real-time polymerase chain reaction
RF: Random Forest
ROC: Receiver-operating characteristics
RPISeq: RNA–Protein Interaction Prediction
SD: Standard deviation
SE: Standard error
sPD-1: Soluble Programmed Death-1
sPD-L1: Soluble Programmed Death-Ligand 1
SVM: Support vector machine
TCGA: The Cancer Genome Atlas
TNBC: Triple-negative breast cancer
