# Supplementary material for: The interplay of the lncRNA AFAP1-AS1 and the soluble immune checkpoint proteins sPD-1/sPD-L1 in breast cancer: tumor–immune cross-talk
Source: Biosci Rep. 2026 Oct 5;46(10):BSR20250198. doi: 10.1042/BSR20250198 (PMC13643020; doi:10.1042/BSR20250198)
Supplement: Supplementary file 1 [file BSR-2025-0198_supp.pdf]

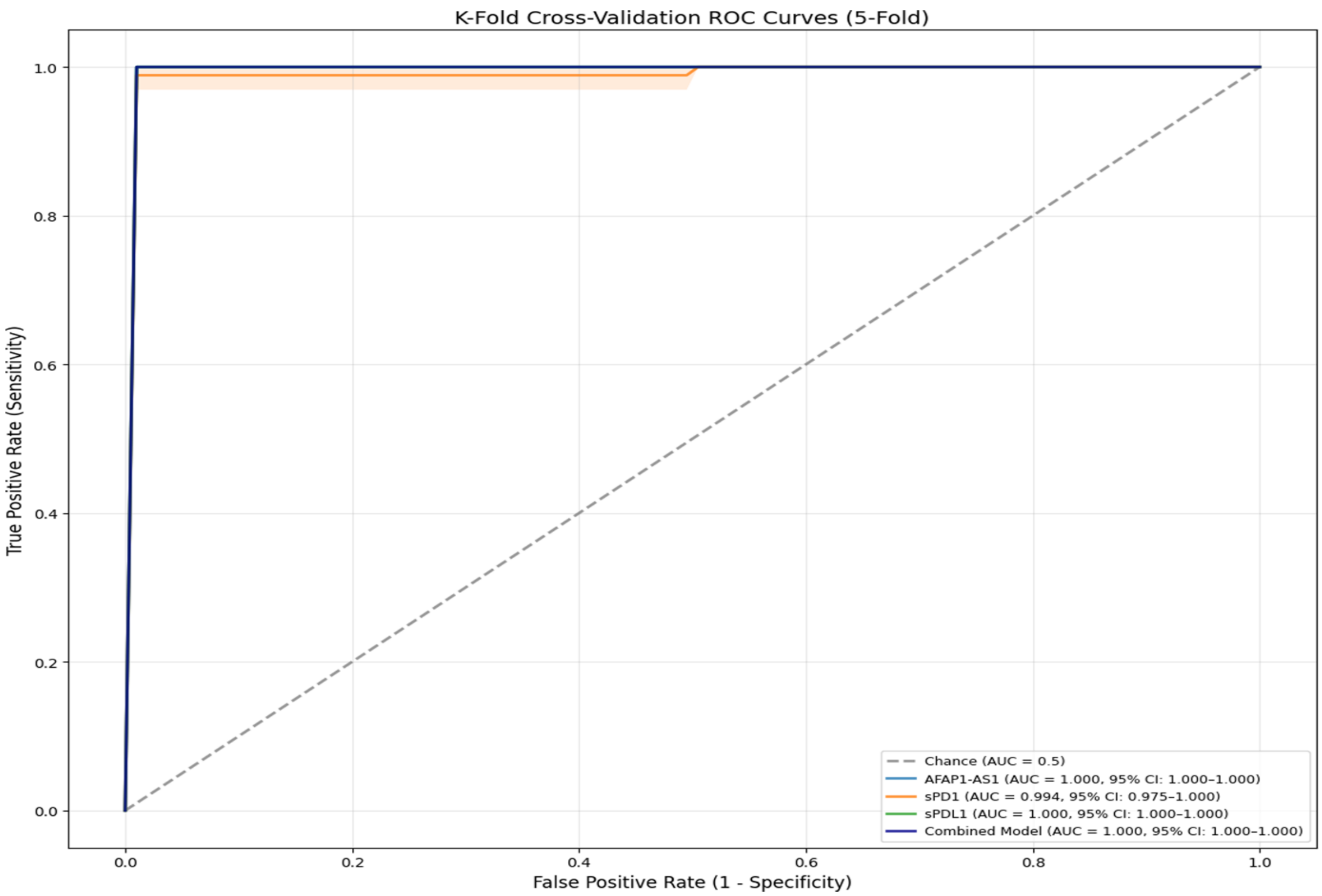

Supplementary Figure S1. K-Fold (5-Fold) Cross-Validated ROC Curves for Discrimination between Cases and Controls.

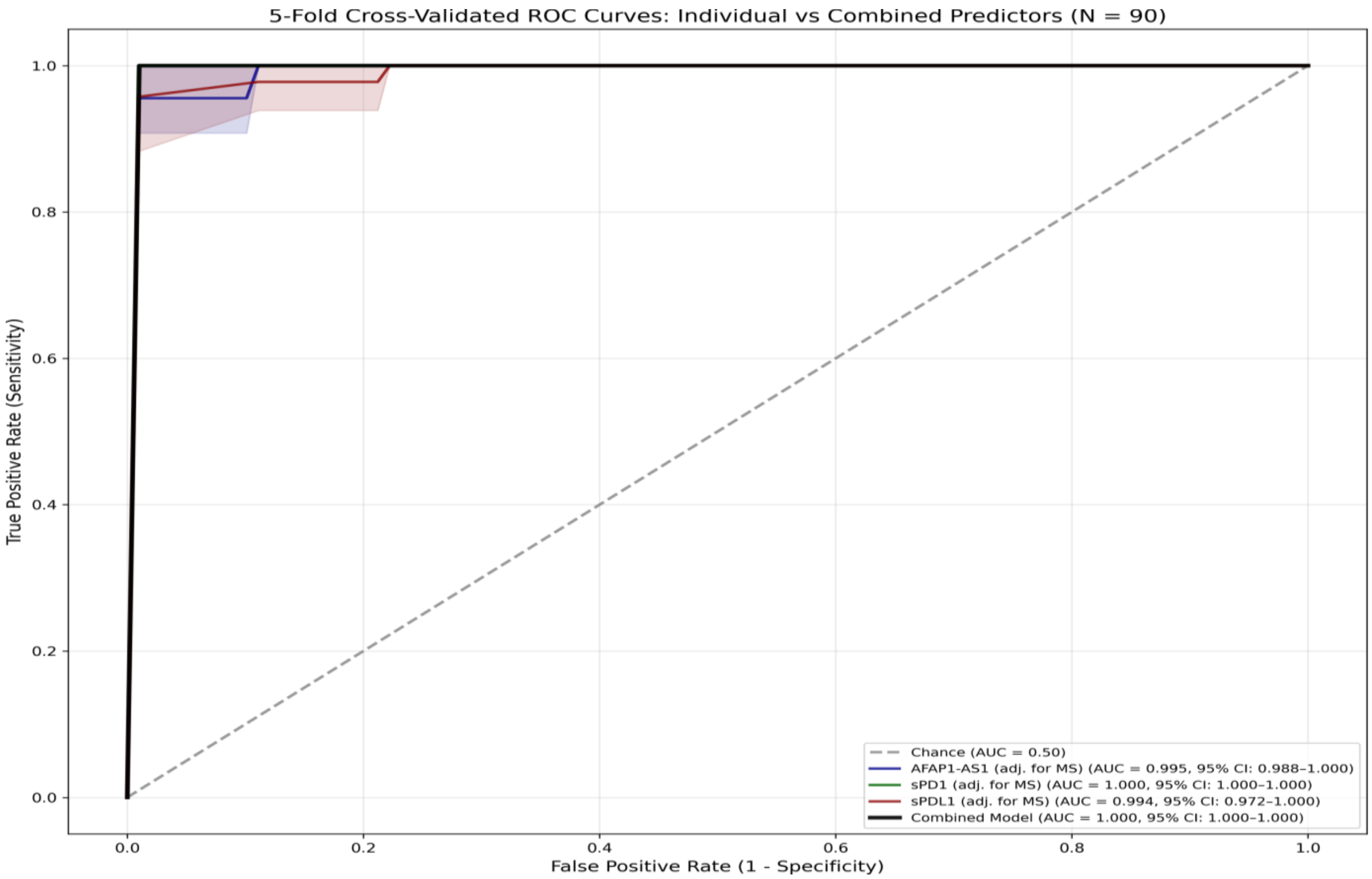

Supplementary Figure S2. K-Fold (5-Fold) Cross-Validated ROC Curves for Discrimination between Cases with Metastatic or Non-Metastatic Disease as Adjusted for the Confounding Effect of Molecular Subtype.

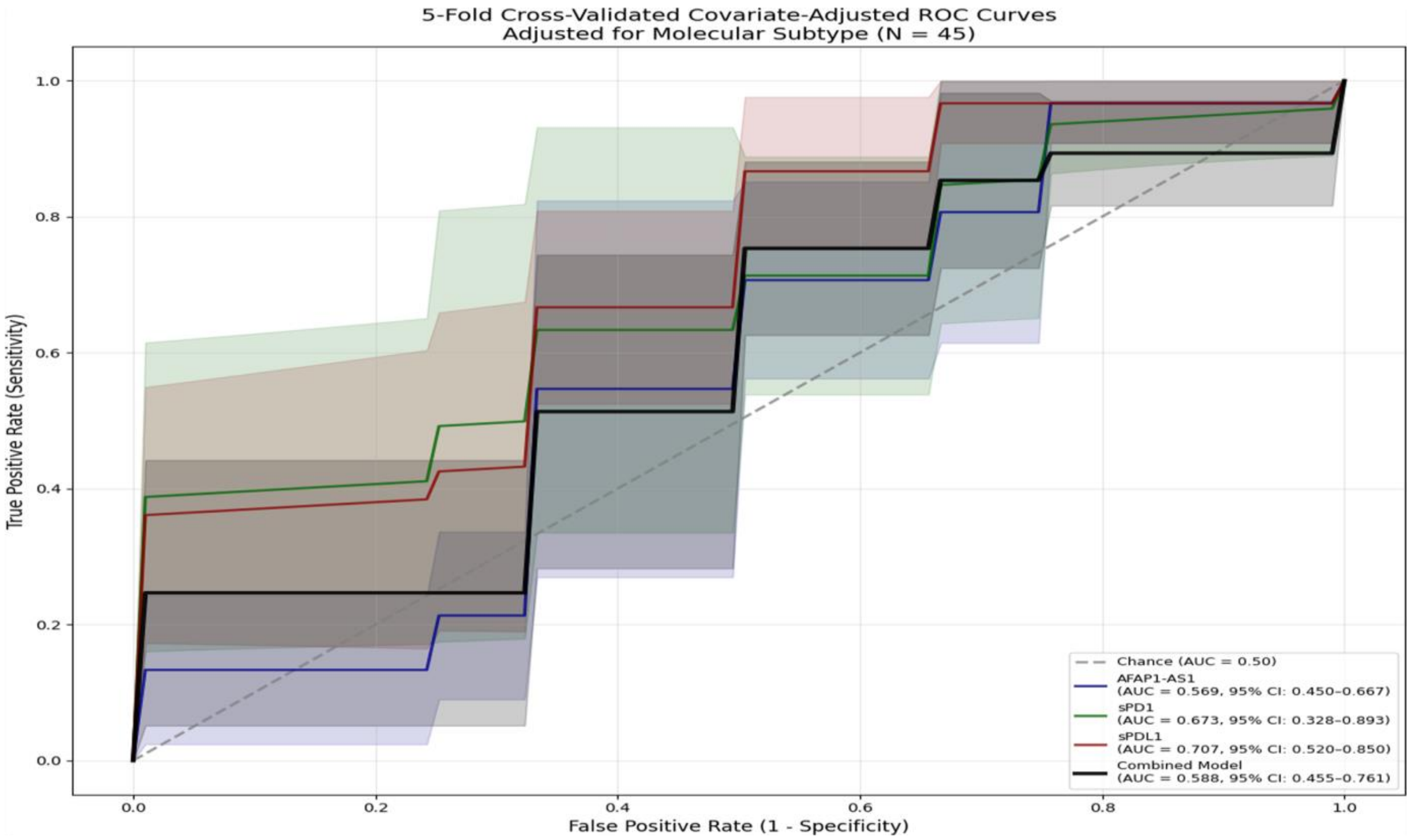

Supplementary Figure S3. K-Fold (5-Fold) Cross-Validated ROC Curves for Discrimination between Cases with Metastatic Disease Who Have Distant or Contralateral-Side Metastasis as Adjusted for the Confounding Effect of Molecular Subtype.

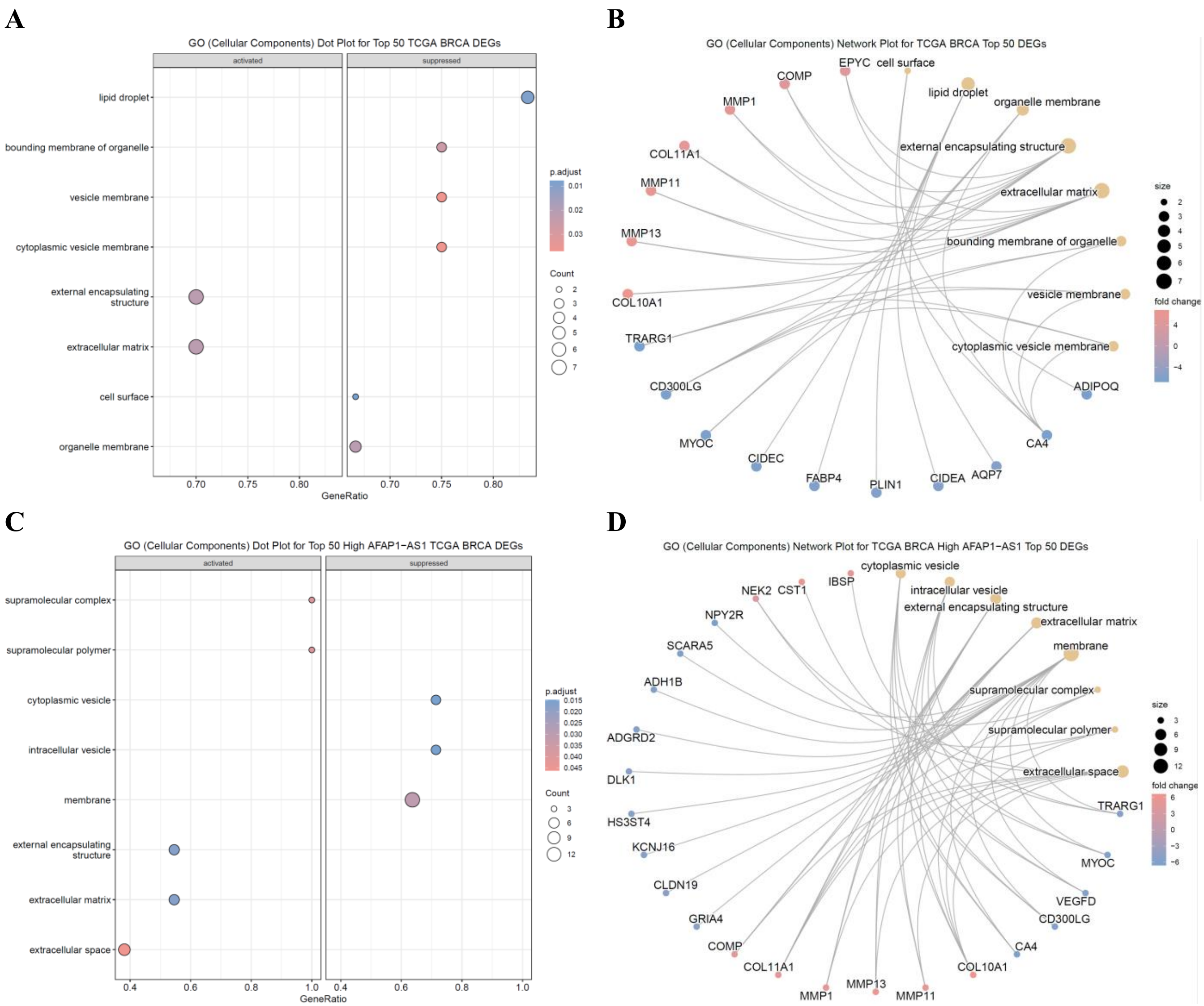

Supplementary Figure S4. Gene set enrichment analysis for AFAP1-AS1 in TCGA BRCA samples.
A. GO (Biological Processes) Dot Plot for Top 50 TCGA BRCA DEGs. B. GO (Biological Processes) Network for Top 50 TCGA BRCA DEGs. C. GO (Biological Processes) Dot Plot for Top 50 TCGA BRCA DEGs (High AFAP1-AS1 Samples). D. GO (Biological Processes) Network for Top 50 TCGA BRCA DEGs (High AFAP1-AS1 Samples).
