# Supplementary material for: The interplay of the lncRNA AFAP1-AS1 and the soluble immune checkpoint proteins sPD-1/sPD-L1 in breast cancer: tumor–immune cross-talk
Source: Biosci Rep. 2026 Oct 5;46(10):BSR20250198. doi: 10.1042/BSR20250198 (PMC13643020; doi:10.1042/BSR20250198)
Supplement: Supplementary file 2 [file BSR-2025-0198_supp1.zip › BSR-2025-0198-s02.pdf]

Table S2-A: K-Fold (5-Fold) Cross-Validated ROC Curves for Discrimination between Cases and Controls

**PART A: INDIVIDUAL PREDICTOR ANALYSIS**
**Analyzing predictor: AFAP1-AS1**
Fold 1: AUC = 1.000, Cutoff = 0.937, Sens = 1.000, Spec = 1.000
Fold 2: AUC = 1.000, Cutoff = 0.977, Sens = 1.000, Spec = 1.000
Fold 3: AUC = 1.000, Cutoff = 0.937, Sens = 1.000, Spec = 1.000
Fold 4: AUC = 1.000, Cutoff = 0.955, Sens = 1.000, Spec = 1.000
Fold 5: AUC = 1.000, Cutoff = 0.953, Sens = 1.000, Spec = 1.000
SUMMARY for AFAP1-AS1:
AUC = 1.000 (95% CI: 1.000–1.000)
Optimal Cutoff = 0.952 (95% CI: 0.937–0.975)
Sensitivity = 1.000 (95% CI: 1.000–1.000)
Specificity = 1.000 (95% CI: 1.000–1.000)
**Analyzing predictor: sPD1**
Fold 1: AUC = 1.000, Cutoff = 0.856, Sens = 1.000, Spec = 1.000
Fold 2: AUC = 1.000, Cutoff = 0.870, Sens = 1.000, Spec = 1.000
Fold 3: AUC = 0.972, Cutoff = 0.905, Sens = 0.944, Spec = 1.000
Fold 4: AUC = 1.000, Cutoff = 0.849, Sens = 1.000, Spec = 1.000
Fold 5: AUC = 1.000, Cutoff = 0.847, Sens = 1.000, Spec = 1.000
SUMMARY for sPD1:
AUC = 0.994 (95% CI: 0.975–1.000)
Optimal Cutoff = 0.865 (95% CI: 0.847–0.902)
Sensitivity = 0.989 (95% CI: 0.950–1.000)
Specificity = 1.000 (95% CI: 1.000–1.000)
**Analyzing predictor: sPDL1**
Fold 1: AUC = 1.000, Cutoff = 0.762, Sens = 1.000, Spec = 1.000

Fold 2: AUC = 1.000, Cutoff = 0.914, Sens = 1.000, Spec = 1.000
Fold 3: AUC = 1.000, Cutoff = 0.679, Sens = 1.000, Spec = 1.000
Fold 4: AUC = 1.000, Cutoff = 0.749, Sens = 1.000, Spec = 1.000
Fold 5: AUC = 1.000, Cutoff = 0.897, Sens = 1.000, Spec = 1.000
SUMMARY for sPDL1:
AUC = 1.000 (95% CI: 1.000–1.000)
Optimal Cutoff = 0.800 (95% CI: 0.686–0.913)
Sensitivity = 1.000 (95% CI: 1.000–1.000)
Specificity = 1.000 (95% CI: 1.000–1.000)
**PART B: COMBINED PREDICTORS ANALYSIS**
**Analyzing Combined Predictors: AFAP1-AS1, sPD1, sPDL1**
Fold 1: AUC = 1.000, Cutoff = 0.948, Sens = 1.000, Spec = 1.000
Fold 2: AUC = 1.000, Cutoff = 0.999, Sens = 1.000, Spec = 1.000
Fold 3: AUC = 1.000, Cutoff = 0.982, Sens = 1.000, Spec = 1.000
Fold 4: AUC = 1.000, Cutoff = 0.993, Sens = 1.000, Spec = 1.000
Fold 5: AUC = 1.000, Cutoff = 0.626, Sensitivity = 1.000, Specificity = 1.000
SUMMARY for Combined Predictors:
AUC = 1.000 (95% CI: 1.000–1.000)
Optimal Cutoff = 0.910 (95% CI: 0.658–0.999)
Sensitivity = 1.000 (95% CI: 1.000–1.000)
Specificity = 1.000 (95% CI: 1.000–1.000)

AUC: area under the curve, *CI*: confidence interval, *Sens*: sensitivity, *Spec*: specificity.

Table S2-B: Summary of K-Fold (5-Fold) Cross-Validated ROC Curves for Discrimination between Cases and Controls

Predictor	AUC (95% CI)	Cutoff (95% CI)	Sensitivity (95% CI)	Specificity (95% CI)
AFAP1-AS1	1.000 (1.000–1.000)	0.952 (0.937–0.975)	1.000 (1.000–1.000)	1.000 (1.000–1.000)
sPD1	0.994 (0.975–1.000)	0.865 (0.847–0.902)	0.989 (0.950–1.000)	1.000 (1.000–1.000)
sPDL1	1.000 (1.000–1.000)	0.800 (0.686–0.913)	1.000 (1.000–1.000)	1.000 (1.000–1.000)
Combined Model	1.000 (1.000–1.000)	0.910 (0.658–0.999)	1.000 (1.000–1.000)	1.000 (1.000–1.000)

CI: confidence interval.

Table S3-A. K-Fold (5-Fold) Cross-Validated ROC Curves for Discrimination between Cases with Metastatic or Non-Metastatic Disease as Adjusted for the Confounding Effect of Molecular Subtype

**PART A: INDIVIDUAL PREDICTOR ANALYSIS (Adjusted for Molecular Subtype)**
**Analyzing predictor: AFAP1-AS1 (Adjusted for Molecular Subtype)**
Fold 1: AUC = 1.000, Cutoff = 0.640, Sens = 1.000, Spec = 1.000
Fold 2: AUC = 1.000, Cutoff = 0.555, Sens = 1.000, Spec = 1.000
Fold 3: AUC = 0.988, Cutoff = 0.716, Sens = 0.889, Spec = 1.000
Fold 4: AUC = 0.988, Cutoff = 0.626, Sens = 0.889, Spec = 1.000
Fold 5: AUC = 1.000, Cutoff = 0.866, Sens = 1.000, Spec = 1.000
SUMMARY for AFAP1-AS1 (Adjusted for Molecular Subtype):
AUC = 0.995 (95% CI: 0.988–1.000)
Optimal Cutoff = 0.681 (95% CI: 0.562–0.851)
Sensitivity = 0.956 (95% CI: 0.889–1.000)
Specificity = 1.000 (95% CI: 1.000–1.000)
**Analyzing predictor: sPD1 (Adjusted for Molecular Subtype)**
Fold 1: AUC = 1.000, Cutoff = 0.883, Sens = 1.000, Spec = 1.000
Fold 2: AUC = 1.000, Cutoff = 0.864, Sens = 1.000, Spec = 1.000
Fold 3: AUC = 1.000, Cutoff = 0.656, Sens = 1.000, Spec = 1.000
Fold 4: AUC = 1.000, Cutoff = 0.645, Sens = 1.000, Spec = 1.000
Fold 5: AUC = 1.000, Cutoff = 0.754, Sens = 1.000, Spec = 1.000
SUMMARY for sPD1 (adjusted for Molecular Subtype):
AUC = 1.000 (95% CI: 1.000–1.000)
Optimal Cutoff = 0.760 (95% CI: 0.646–0.881)
Sensitivity = 1.000 (95% CI: 1.000–1.000)
Specificity = 1.000 (95% CI: 1.000–1.000)
**Analyzing predictor: sPDL1 (Adjusted for Molecular Subtype)**

Fold 1: AUC = 1.000, Cutoff = 0.506, Sens = 1.000, Spec = 1.000
Fold 2: AUC = 1.000, Cutoff = 0.754, Sens = 1.000, Spec = 1.000
Fold 3: AUC = 0.969, Cutoff = 0.914, Sens = 0.778, Spec = 1.000
Fold 4: AUC = 1.000, Cutoff = 0.567, Sens = 1.000, Spec = 1.000
Fold 5: AUC = 1.000, Cutoff = 0.623, Sens = 1.000, Spec = 1.000
SUMMARY for sPDL1 (adjusted for Molecular Subtype):
AUC = 0.994 (95% CI: 0.972–1.000)
Optimal Cutoff = 0.673 (95% CI: 0.512–0.898)
Sensitivity = 0.956 (95% CI: 0.800–1.000)
Specificity = 1.000 (95% CI: 1.000–1.000)
**PART B: COMBINED PREDICTORS ANALYSIS (Adjusted for Molecular Subtype)**
**Combining all predictors: AFAP1-AS1, sPD1, sPDL1(Adjusted for Molecular subtype):**
Fold 1: AUC = 1.000, Cutoff = 0.893, Sens = 1.000, Spec = 1.000
Fold 2: AUC = 1.000, Cutoff = 0.881, Sens = 1.000, Spec = 1.000
Fold 3: AUC = 1.000, Cutoff = 0.629, Sens = 1.000, Spec = 1.000
Fold 4: AUC = 1.000, Cutoff = 0.776, Sens = 1.000, Spec = 1.000
Fold 5: AUC = 1.000, Cutoff = 0.939, Sens = 1.000, Spec = 1.000
SUMMARY for Combined Model (Adjusted for Molecular subtype):
AUC = 1.000 (95% CI: 1.000–1.000)
Optimal Cutoff = 0.824 (95% CI: 0.644–0.934)
Sensitivity = 1.000 (95% CI: 1.000–1.000)
Specificity = 1.000 (95% CI: 1.000–1.000)

AUC: area under the curve, *CI*: confidence interval, *Sens*: sensitivity, *Spec*: specificity.

Table S3-B. Summary of K-Fold (5-Fold) Cross-Validated ROC Curves for Discrimination between Cases with Metastatic or Non-Metastatic Disease as Adjusted for the Confounding Effect of Molecular Subtype

Predictor*	AUC (95% CI)	Cutoff (95% CI)	Sensitivity (95% CI)	Specificity (95% CI)
AFAP1-AS1	0.995 (0.988–1.000)	0.681 (0.562–0.851)	0.956 (0.889–1.000)	1.000 (1.000–1.000)
sPD1	1.000 (1.000–1.000)	0.760 (0.646–0.881)	1.000 (1.000–1.000)	1.000 (1.000–1.000)
sPDL1	0.994 (0.972–1.000)	0.673 (0.512–0.898)	0.956 (0.800–1.000)	1.000 (1.000–1.000)
Combined Model	1.000 (1.000–1.000)	0.824 (0.644–0.934)	1.000 (1.000–1.000)	1.000 (1.000–1.000)

*. All predictors are adjusted for the Molecular Subtype.
CI: confidence interval.

Table S4-A: K-Fold (5-Fold) Cross-Validated ROC Curves for Discrimination between Cases with Metastatic Disease Who Have Distant or Contralateral-Side Metastasis as Adjusted for the Confounding Effect of Molecular Subtype

**PART A: INDIVIDUAL PREDICTOR ANALYSIS (Adjusted for Molecular Subtype)**
**Analyzing: AFAP1-AS1 (Adjusted for Molecular subtype)**
Fold 1: AUC = 0.667, Cutoff = 0.545, Sens = 1.000, Spec = 0.333
Fold 2: AUC = 0.667, Cutoff = 0.562, Sens = 0.833, Spec = 0.667
Fold 3: AUC = 0.611, Cutoff = 0.395, Sens = 0.833, Spec = 0.667
Fold 4: AUC = 0.450, Cutoff = 0.566, Sens = 0.800, Spec = 0.500
Fold 5: AUC = 0.450, Cutoff = 0.616, Sens = 1.000, Spec = 0.250
SUMMARY for AFAP1-AS1 (Adjusted for Molecular subtype):
AUC = 0.569 (95% CI: 0.450–0.667)
Optimal Cutoff = 0.537 (95% CI: 0.410–0.611)
Sensitivity = 0.893 (95% CI: 0.803–1.000)
Specificity = 0.483 (95% CI: 0.258–0.667)
**Analyzing: sPD1 (Adjusted for Molecular subtype)**
Fold 1: AUC = 0.750, Cutoff = 0.678, Sens = 0.500, Spec = 1.000
Fold 2: AUC = 0.833, Cutoff = 0.688, Sens = 0.667, Spec = 1.000
Fold 3: AUC = 0.583, Cutoff = 0.595, Sens = 0.667, Spec = 0.667
Fold 4: AUC = 0.300, Cutoff = 0.419, Sens = 0.800, Spec = 0.250
Fold 5: AUC = 0.900, Cutoff = 0.658, Sens = 1.000, Spec = 0.750
SUMMARY for sPD1 (Adjusted for Molecular subtype):
AUC = 0.673 (95% CI: 0.328–0.893)
Optimal Cutoff = 0.608 (95% CI: 0.436–0.687)
Sensitivity = 0.727 (95% CI: 0.517–0.980)
Specificity = 0.733 (95% CI: 0.292–1.000)
**Analyzing: sPDL1 (Adjusted for Molecular subtype)**

Fold 1: AUC = 0.500, Cutoff = 0.541, Sens = 0.667, Spec = 0.667
Fold 2: AUC = 0.861, Cutoff = 0.589, Sens = 0.667, Spec = 1.000
Fold 3: AUC = 0.722, Cutoff = 0.562, Sens = 0.833, Spec = 0.667
Fold 4: AUC = 0.700, Cutoff = 0.526, Sens = 1.000, Spec = 0.500
Fold 5: AUC = 0.750, Cutoff = 0.630, Sens = 1.000, Spec = 0.500
SUMMARY for sPDL1 (Adjusted for Molecular subtype):
AUC = 0.707 (95% CI: 0.520–0.850)
Optimal Cutoff = 0.570 (95% CI: 0.528–0.626)
Sensitivity = 0.833 (95% CI: 0.667–1.000)
Specificity = 0.667 (95% CI: 0.500–0.967)
**Analyzing: Combined Model (Adjusted for Molecular subtype)**
Predictors: AFAP1-AS1, sPD1, sPDL1
Fold 1: AUC = 0.500, Cutoff = 0.541, Sens = 0.667, Spec = 0.667
Fold 2: AUC = 0.778, Cutoff = 0.610, Sens = 0.667, Spec = 1.000
Fold 3: AUC = 0.611, Cutoff = 0.422, Sens = 0.833, Spec = 0.667
Fold 4: AUC = 0.450, Cutoff = 0.773, Sens = 0.200, Spec = 1.000
Fold 5: AUC = 0.600, Cutoff = 0.619, Sens = 1.000, Spec = 0.500
SUMMARY for Combined Model (Adjusted for Molecular subtype):
AUC = 0.588 (95% CI: 0.455–0.761)
Optimal Cutoff = 0.593 (95% CI: 0.433–0.758)
Sensitivity = 0.673 (95% CI: 0.247–0.983)
Specificity = 0.767 (95% CI: 0.517–1.000)

AUC: area under the curve, *CI*: confidence interval, *Sens*: sensitivity, *Spec*: specificity.

Table S4-B: Summary of K-Fold (5-Fold) Cross-Validated ROC Curves for Discrimination between Cases with Metastatic Disease Who Have Distant or Contralateral-Side Metastasis as Adjusted for the Confounding Effect of Molecular Subtype

Predictor*	AUC (95% CI)	Cutoff (95% CI)	Sensitivity (95% CI)	Specificity (95% CI)
AFAP1-AS1	0.569 (0.450–0.667)	0.537 (0.410–0.611)	0.893 (0.803–1.000)	0.483 (0.258–0.667)
sPD1	0.673 (0.328–0.893)	0.608 (0.436–0.687)	0.727 (0.517–0.980)	0.733 (0.292–1.000)
sPDL1	0.707 (0.520–0.850)	0.57 (0.528–0.626)	0.833 (0.667–1.000)	0.667 (0.500–0.967)
Combined Model	0.588 (0.455–0.761)	0.593 (0.433–0.758)	0.673 (0.247–0.983)	0.767 (0.517–1.000)

*. All predictors are adjusted for the Molecular Subtype.
CI: confidence interval.

Supplementary Table S8. Proteins that bind to AFAP1-AS1 from RNAct.

RNA	Protein	Prediction (catRAPID)	Interaction (ENCODE eCLIP)				
**Transcript**	**Ensembl Transcript ID**	**Gene**	**UniProt Accession**	**Length**	**Protein Status**	**Prediction Score**	**p Value**
AFAP1-AS1-201	ENST00000608442	SF3B1	O75533	1304 aa	Known RBP eCLIP	9.36	9.00E-06
AFAP1-AS1-201	ENST00000608442	AARS	P49588	968 aa	Known RBP eCLIP	9.85	3.00E-06
AFAP1-AS1-201	ENST00000608442	UTP3	Q9NQZ2	479 aa	Known RBP eCLIP	11.35	1.00E-06
AFAP1-AS1-201	ENST00000608442	TARDBP	Q13148	414 aa	Known RBP eCLIP	9.09	1.00E-06
AFAP1-AS1-201	ENST00000608442	GTF2F1	P35269	517 aa	Known RBP eCLIP	10.73	5.00E-08
AFAP1-AS1-201	ENST00000608442	DROSHA	Q9NRR4	1374 aa	Known RBP eCLIP	9.82	5.00E-06
AFAP1-AS1-201	ENST00000608442	SLTM	Q9NWH9	1034 aa	Known RBP eCLIP	10.89	1.00E-06
AFAP1-AS1-201	ENST00000608442	UCHL5	Q9Y5K5	329 aa	Known RBP eCLIP	10.45	4.00E-07
AFAP1-AS1-201	ENST00000608442	PCBP1	Q15365	356 aa	Known RBP eCLIP	8.86	9.00E-09
AFAP1-AS1-201	ENST00000608442	YWHAG	P61981	247 aa	Known RBP eCLIP	10.07	2.00E-06
AFAP1-AS1-201	ENST00000608442	NIPBL	Q6KC79	2804 aa	eCLIP	4.22	8.00E-08
AFAP1-AS1-201	ENST00000608442	CSTF2T	Q9H0L4	616 aa	Known RBP eCLIP	8.95	1.00E-07
AFAP1-AS1-201	ENST00000608442	YBX3	P16989	372 aa	Known RBP eCLIP	9.29	1.00E-06
AFAP1-AS1-201	ENST00000608442	LIN28B	Q6ZN17	250 aa	Known RBP eCLIP	8.57	3.00E-08
AFAP1-AS1-201	ENST00000608442	WDR43	Q15061	677 aa	Known RBP eCLIP	12.27	3.00E-07
AFAP1-AS1-201	ENST00000608442	UPF1	Q92900	1129 aa	Known RBP eCLIP	8.95	1.00E-06
AFAP1-AS1-201	ENST00000608442	FUS	P35637	526 aa	Known RBP eCLIP	7.28	1.00E-06
AFAP1-AS1-201	ENST00000608442	AKAP8L	Q9ULX6	646 aa	Known RBP eCLIP	9.23	9.00E-07
AFAP1-AS1-201	ENST00000608442	SAFB2	Q14151	953 aa	Known RBP eCLIP	11.69	2.00E-06
AFAP1-AS1-201	ENST00000608442	IGF2BP2	Q9Y6M1	599 aa	Known RBP eCLIP	10.29	5.00E-06
AFAP1-AS1-201	ENST00000608442	DDX3X	O00571	662 aa	Known RBP eCLIP	9.28	6.00E-06
